# EEG neurofeedback for the treatment of neuropathic pain in the elderly—a mechanistic review

**DOI:** 10.1007/s11357-025-01848-7

**Published:** 2025-08-30

**Authors:** James Chmiel, Marta Kopańska, Jerzy Leszek, Julia Trojniak, Tomas Ros

**Affiliations:** 1 Institute of Physical Culture Sciences, University of Szczecin, Szczecin, Poland; 2 Department of Medical Psychology, Faculty of Medicine, University of Rzeszów, Rzeszów, Poland; 3 Department and Clinic of Psychiatry, Wrocław Medical University, Wrocław, Poland; 4 Student Research Club “Reh-Tech”, Medical College of Rzeszow University, Rzeszów, Poland; 5 Department of Clinical Neuroscience, University of Geneva, Geneva, Switzerland; 6 Center for Biomedical Imaging (CIBM), Lausanne, Switzerland

**Keywords:** Neurofeedback, EEG, Neuropathic pain, qEEG, EEG, Biofeedback

## Abstract

Neuropathic pain (NP) is a complex pain disorder that constitutes a significant problem in the aging population, impacting quality of life and everyday functioning. In the quest to develop effective treatments, much research effort has been made to understand brain activity in people with NP, revealing a number of disordered electroencephalogram (EEG) patterns. This information can then be used to inform neurofeedback therapy, a novel approach that involves volitionally training brain activity in a closed loop. In this review of the existent literature we had three main objectives: (1) to summarize the reported EEG signatures of NP, (2) to evaluate the therapeutic efficacy of neurofeedback in the treatment of NP, and (3) to present the potential mechanisms of neurofeedback action in NP. Consequently, literature searches were conducted on the PubMed/Medline, Research Gate, and Cochrane databases. We identified 18 studies that examined resting-state EEG patterns in NP, and seven studies that investigated EEG-based neurofeedback in NP. Most biomarker studies of NP showed typical EEG patterns consisting of excess theta activity and decreased alpha activity. Neurofeedback study outcomes were largely promising in terms of treatment efficacy, but their quality was low. In turn, based on these results, we proposed hypothesis-based neurofeedback protocols and discussed the potential mechanisms of neurofeedback in the treatment of NP, including why this treatment option may be beneficial in the elderly population. Neurofeedback is a promising treatment option for NP, but caution should be exercised in interpreting the results due to the low number and methodological quality of research studies. A larger body of research studies points to common patterns of EEG abnormality in NP, which could be directly targeted with neurofeedback. The main advantage of this therapeutic approach is that it has no side effects and may be considered a valuable form of treatment in more frail populations such as the elderly.

## Introduction

### Defining neuropathic pain (NP) and its scope

Neuropathic pain (NP), a complex and often perplexing condition, presents a significant clinical challenge in the field of pain management. Unlike nociceptive pain, which arises from direct activation of pain receptors in response to tissue damage, NP stems from dysfunction or damage to the nervous system itself [1]. This enigmatic form of chronic pain is characterized by abnormal sensory experiences, including burning, shooting, or electric shock-like sensations, tingling, numbness, and heightened sensitivity to touch or temperature changes [2]. NP can occur in association with various diseases and conditions, for example in multiple sclerosis [3], diabetic neuropathy [4], postherpetic neuralgia [5], spinal cord injury [6], amputation [7], Parkinson’s disease [8], and stroke [9]. It is a relatively common disease, affecting approximately 3.2 to 10.3% of the population [10], which accounts for 20 to 25% of all people with chronic pain [11, 12]. In each of the previously mentioned conditions, the prevalence of neuropathic pain is different (Table 1).

**Table 1:** Percentage range of occurrence of neuropathic pain (NP) in particular conditions and diseases

Condition/disease	Occurrence of NP	References
Painful diabetic polyneuropathy (DPN)	14–26%	[13–15]
Post-herpetic neuralgia (PHN)	8–10%	[16, 17]
Cancer	17–19%	[18–20]
Neuropathic pain related to surgery	10–50%	[21–26]
Multiple sclerosis (SM)	20–30%	[27–30]
Spinal cord injury	30–40%	[31, 32]
Stroke	5–11%	[33, 34]
Parkinson’s disease	15.3%	[35]

Neuropathic pain is more common in patients over the age of 60 than in younger patients and in women than in men [11, 12, 36–39].

### Pathophysiology of NP: peripheral and central mechanisms

The pathophysiological mechanisms underlying NP are complex, involving interactions between peripheral nerves, spinal cord pathways, and the brain. NP often occurs due to direct injury or damage to the nerves [40]. This can result from various causes such as trauma [41], compression [42], inflammation [43], infection [42], or metabolic disorders [42]. Nerve damage disrupts the normal transmission of pain signals and can lead to spontaneous firing or hyperexcitability of damaged nerves [44]. In response to nerve injury or inflammation, the peripheral nerves become sensitized, leading to heightened responsiveness to stimuli. This phenomenon is known as peripheral sensitization. It involves the release of various chemical mediators, such as pro-inflammatory cytokines, chemokines, and growth factors, which sensitize the nerve fibers and enhance their sensitivity to stimuli. This increased sensitivity contributes to the development of spontaneous pain and allodynia (pain in response to normally non-painful stimuli) or hyperalgesia (exaggerated pain response to a painful stimulus) [45].

NP is also associated with central sensitization, which involves changes in the central nervous system (CNS), particularly in the spinal cord and brain [46]. Persistent input from damaged nerves leads to an amplification of pain signals within the CNS, causing an increased response to painful stimuli. Central sensitization can result in widespread pain, hypersensitivity, and the expansion of pain perception beyond the original site of injury [47].

### Role of neuroinflammation and maladaptive plasticity

Inflammatory processes play a significant role in NP. Nerve injury triggers the release of inflammatory molecules and immune cells, leading to neuroinflammation. Inflammatory mediators, such as cytokines, chemokines, and prostaglandins, contribute to neuronal hyperexcitability, sensitization of pain pathways, and the recruitment of immune cells that further perpetuate the inflammatory response. Neuroinflammation can lead to long-term changes in pain processing and the maintenance of chronic pain states [48]. Persistent NP can induce maladaptive plasticity in the nervous system. This includes structural and functional changes in neuronal circuits, such as altered synaptic connectivity and reorganization of pain pathways. Maladaptive plasticity can contribute to resistance to treatment [49].

### Current management strategies for NP

Managing NP can be challenging, but a multimodal approach that combines various treatment strategies can help alleviate symptoms and improve the quality of life for individuals affected by this condition.

Several classes of medications can be used to target NP. These include antidepressants (such as tricyclic antidepressants and selective serotonin-norepinephrine reuptake inhibitors) [50], anticonvulsants (such as gabapentin and pregabalin) [51], topical medications (such as lidocaine patches) [52], and opioids (in severe cases) [53]. These medications work by modulating pain signals, reducing nerve excitability, and altering neurotransmitter levels.

Electrical stimulation of specific nerves or regions of the spinal cord can provide pain relief for some individuals. Transcutaneous electrical nerve stimulation (TENS) and spinal cord stimulation (SCS) are two common techniques used in neurostimulation. TENS involves the use of a portable device to deliver low-voltage electrical currents to the skin over the painful area [54], while SCS involves implanting electrodes near the spinal cord to deliver electrical impulses that interfere with pain signals [55]. Physical therapy and rehabilitation programs can help improve physical functioning and reduce pain in neuropathic conditions. Techniques such as exercise therapy, stretching, and strengthening exercises can enhance muscle strength, promote flexibility, and improve overall mobility [56]. Physical therapists may also use modalities like heat or cold therapy, ultrasound, or TENS to manage pain symptoms [56].

### NP in the elderly: specific considerations and challenges

In the elderly population, NP is prevalent and a significant contributor to morbidity and suffering. Data regarding the elderly are more scarce, but reports indicate that the prevalence of this disease in this population may be as high as 32% [57–59]. However, measuring the occurrence of NP in older adults is difficult; because of cognitive decline and other conditions, older adults frequently underreport pain, especially to primary care physicians [11]. Due to the increased occurrence of numerous disorders that cause neuropathy, older persons are more susceptible to NP. These include spinal degenerative disease, radiculopathies, many malignancies, and chemotherapy. NP is also linked with diabetes (diabetic neuropathy), herpes zoster (postherpetic neuralgia), stroke (central neuropathic pain), and limb amputations (phantom limb pain) [60]. Furthermore, structural and biochemical alterations associated with aging include the loss of neurons in the central nervous system, an increase in aberrant or degenerating fibers, a slower rate of conduction, changes to endogenous inhibition, and a decline in the function of neurotransmitters. Older adults’ altered perception of NP is related to these structural changes [61].

People with NP sometimes attribute their chronic pain to mood issues and sleep disruptions. Patients with NP frequently have lower life satisfaction, both as a result of the disease’s symptoms and their effects on quality of life. The quality of life may be impacted by NP to the same extent as with other chronic conditions, such as poorly managed diabetes mellitus or coronary artery disease. Depression is a frequent outcome, especially when it’s linked to more intense pain. When it comes to older adults, untreated chronic pain is linked to a higher risk of falling, poor sleep, social isolation, and functional decline [61].

Elderly NP is frequently not adequately treated pharmacologically [62]. Comorbidities may affect how older adults should manage their chronic pain and its aftereffects [61]. A comprehensive assessment of the kinds, timing, and dose of pharmaceutical therapy is necessary for certain clinical disorders, such as heart failure or chronic renal disease. The elderly and people with various illnesses typically need to take multiple chronic drugs, which may interact with pain relief prescriptions. Physicians need to pay close attention to this because polypharmacy is linked to a number of unfavorable outcomes, such as death, increased rates of hospitalization, and longer hospital stays [61].

### Limitations of conventional therapies and rationale for exploring alternatives

Conventional therapies for neuropathic pain, such as pharmacotherapy, are often insufficient to provide long-term relief to patients. This is due to the complex nature of neuropathic pain, which includes both physiological and psychological components [63]. One of the main challenges is the limited effectiveness of pharmacotherapy—painkillers, including opioids and antidepressants, often do not bring significant improvement, and their long-term use can lead to serious side effects and the development of tolerance [63].

Another difficulty is the complexity of the mechanisms of neuropathic pain. This pain is associated with abnormal activity in the central nervous system, which is why standard treatments often fail [2]. In addition, treatment is complicated by individual differences in patient response to the therapies used, which means that there is no single, universal approach that is effective for all [2]. Due to these limitations, there is growing interest in alternative treatments such as neurofeedback.

### Neurofeedback (NF) as a potential therapeutic approach

Neurofeedback (NF), also known as EEG biofeedback, harnesses the brain’s inherent capacity for self-regulation of brainwave activity to induce neuroplasticity. It is a non-invasive and drug-free approach that uses a closed loop to measure and analyze brain wave activity [64]. Brain waves are analyzed using an electroencephalogram (EEG), which records brain wave activity. This tool is commonly used in neurology to diagnose epilepsy, but is currently being studied for its use in many neurological and psychiatric conditions [65]. Neurofeedback training is also being implemented in many of these conditions to assess its effectiveness as a non-invasive therapy. By providing real-time feedback on brainwave patterns through visual or auditory cues, neurofeedback empowers individuals to modulate their brain activity and achieve more optimal states of functioning [66]. This self-regulation involves learning to modulate specific EEG frequencies, such as alpha, beta, theta, and gamma oscillations, which play crucial roles in pain perception, cognitive processes, and emotional regulation [67]. Neurofeedback training is often used to address various neurological and psychological conditions, including autism spectrum disorders (ASD) [68], attention-deficit/hyperactivity disorder (ADHD) [69], anxiety disorders [70], depression [71], insomnia [72], migraines [73], and post-traumatic stress disorder (PTSD) [74]. It is believed that by providing feedback and allowing individuals to learn self-regulation of their brain activity, neurofeedback can help improve symptoms and enhance overall brain function.

One of the key principles of neurofeedback is neuroplasticity, the brain’s ability to reorganize and form new neural connections. With repeated practice and training, neurofeedback aims to promote positive changes in brain functioning and help individuals achieve more optimal brainwave patterns. By training the brain to self-regulate, neurofeedback may have lasting effects even after the training sessions are completed.

### Current gaps and objectives of this review

To date, there have been several comprehensive evaluations exploring the efficacy of neurofeedback as a therapeutic intervention for various pain conditions [75–78]. While these reviews have provided evidence supporting the effectiveness of neurofeedback, they have generally encompassed a broad range of pain types, lacking specificity in the context of NP. Additionally, the underlying mechanisms through which neurofeedback exerts its influence on pain outcomes have not been thoroughly elucidated.

In light of these gaps, this review aims to address these issues by presenting a three-part analysis. Firstly, it will encompass a comprehensive overview of studies investigating EEG activity in individuals with NP, providing a synthesis of the findings and aiming to develop a novel neurofeedback protocol based on the neurophysiological evidence. Secondly, it will focus on studies that have specifically examined the application of neurofeedback in the management of NP, summarizing the key findings and outcomes. Finally, the review will discuss the potential mechanisms of action of neurofeedback in the treatment of NP. Additionally, we will mention the advantages of neurofeedback when used in elderly people with NP.

## Methods

### Data sources and search strategy

To ensure comprehensive and reliable results, we conducted a literature search in several databases: PubMed/Medline, ResearchGate, and Cochrane. These databases were selected based on their high quality, accessibility, and broad coverage, which allowed for obtaining relevant clinical trial data in the field of neurofeedback and neuropathic pain (NP). The search was conducted using a set of defined keywords, including: “neurofeedback,” “neuropathic pain,” “neurogenic,” “EEG,” “electroencephalography,” “electrophysiological,” and “neuralgia.” The search was limited to these phrases because they were highly relevant to the analyzed topic.

### Study selection criteria

Our review only included clinical studies that met specific quality and methodological criteria. Only studies published between January 1999 and May 2024 were included. The time period of 1999–2024 was chosen because it represents the period in which neurofeedback as a therapeutic method gained greater attention in the scientific literature and also represented a period in which EEG and neurofeedback techniques were significantly developed in terms of methodology and technology. Moreover, since 1999, studies began to appear that provided more reliable data in the field of neuropathic pain treatment, so this period was considered the most appropriate for analysis.

### Study exclusion criteria and screening process

In accordance with the aim of this review, all studies that did not address the treatment of neuropathic pain with neurofeedback were excluded. Within this exclusion, all articles that were reviews, systematic reviews, or meta-analyses were not included. Furthermore, studies published in languages ​​other than English were excluded, because the main aim was to collect the latest, internationally recognized studies that could provide the most relevant evidence on the effectiveness of neurofeedback in the treatment of NP.

Additionally, studies that did not meet the criteria of methodological quality, such as lack of a control group, inappropriate EEG analysis method, or too small a study sample that could affect the reliability of the results, were excluded.

#### Title and abstract screening

Each reviewer independently evaluated the titles and abstracts of the records that were available to determine which research fulfilled the inclusion criteria. At this point, the primary focus of the screening criteria was how neurofeedback affected NP and resting state EEG in NP.

#### Full-text assessment

After titles and abstracts were first screened, the chosen papers were subjected to a thorough full-text assessment. The reviewers meticulously examined each article to ensure that it satisfied the eligibility requirements, with a particular focus on making sure the research were clinical trials carried out in English and published between January 1999 and May 2024.

#### Research quality and data analysis

Each of the selected studies was assessed for methodological quality, including sample size, use of a control group, level of detail in describing the EEG methodology, and data analysis. We also assessed any limitations of the individual studies, including failure to control for confounding variables such as medication use or other comorbidities that could have influenced the results. All results were then collated and analyzed for consistency, as well as for the identification of common patterns of EEG activity associated with neuropathic pain.

## Results of studies on EEG in NP

**Fig. 1:**
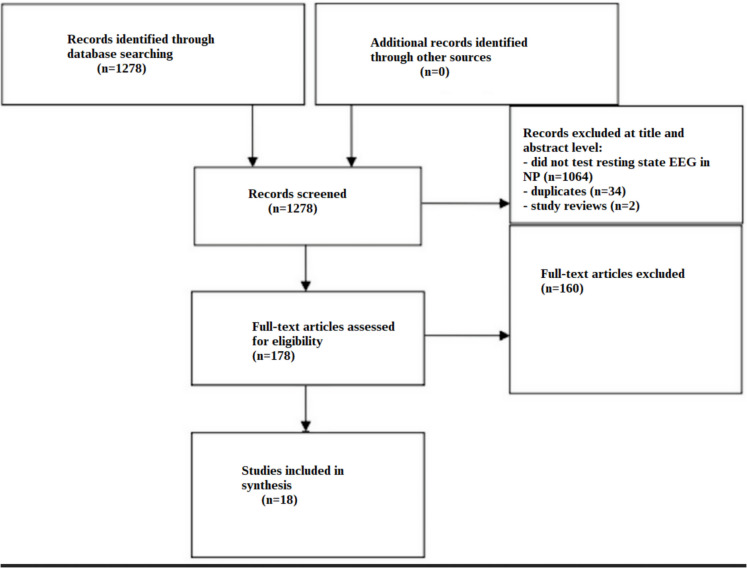
First flow chart depicting the different phases of the review

The second screening process is depicted in a flow chart (Fig. 1). Initially, utilizing search techniques across many databases, we found 1278 studies. After examining the titles and abstracts of these studies, 1100 were disqualified; 1064 of them did not test resting state EEG in NP, 34 of them were duplicates, and 2 of them were research reviews. The final 178 studies were then subjected to a thorough full-text evaluation. Upon careful examination of the texts, it was determined that 18 articles satisfied the requirements for inclusion. The studies on EEG in NP [79–95, 134] were published between 2015 and 2022.

### Studies on EEG in NP—an overview

EEG studies in patients with neuropathic pain focus on analyzing changes in brain activity related to different types of pain, including pain from spinal cord injury, cancer-related neuropathy, pain in multiple sclerosis, and other chronic neuropathic pains. The use of various frequency ranges such as delta, theta, alpha, beta, and gamma allows for a detailed assessment of brain activity patterns in response to pain. These studies also examine changes in EEG power, shifts in dominant frequencies, and differences in brain reactivity depending on whether the eyes are open or closed. The results of these studies provide valuable insights into the neurophysiological mechanisms of neuropathic pain; however, they are often characterized by small sample sizes, the absence of control groups in some cases, and the exclusion of confounding variables such as medication use, which may affect EEG results. While the studies provide interesting findings, more research with control groups and more rigorous methodologies are needed to enhance the reliability and applicability of the results in clinical practice. The following table presents detailed findings from various EEG studies in the context of neuropathic pain. A comprehensive summary of articles on EEG in NP is summarized in Table 2.

**Table 2:** Comparison of EEG results in the context of neuropathic pain: analysis of the frequency and quality of studies

Study	Sample Size	Age	Pain type	EEG conditions	Frequency bands analyzed	Key findings	Electrode location/specific findings	Methodological strengths	Methodological weaknesses	Potential impact on reliability
Sarnthein et al. (2006) [79]	15	60	Neurogenic pain	Eyes open/closed	Theta, alpha, beta, gamma	Higher power across 2–25 Hz; dominant peak shifted to lower frequencies	Highest power in posterior electrodes, significant differences in low alpha band (7–9 Hz), increased coupling between theta and beta	Small sample size, cross-sectional design	Limited generalizability due to small sample	Small sample impacts generalizability; power shifts observed in key brain regions suggest neurophysiological alterations due to pain
Boord et al. (2008) [80]	16	35–46	Neuropathic pain (SCI)	Eyes open/closed	Theta, alpha	Shift to lower frequencies in pain group, significant reductions in peak frequency	Significant reductions at 12 sites	Moderate sample size, control group	No longitudinal data, small sample	Limited control for confounding factors may affect conclusions on frequency shifts
Jensen et al. (2013) [81]	38	51.24	SCI + neuropathic pain	Eyes closed	Theta, alpha, beta	More theta and less alpha activity in pain group	Significant effects at frontal sites for alpha, relative theta at P3, O1, O2	Larger sample size, multiple electrode sites	No major control over other pain influences (e.g., medication)	Strong analysis with clear localization of alpha and theta changes; larger sample improves reliability
Stern et al. (2006) [82]	16	63	Chronic neurogenic pain	Eyes closed	Theta, alpha, beta	Increased theta and beta activity; widespread cortical synchronization	Significant activation in posterior insula, anterior cingulate, DLPFC	Small sample, resting state EEG	Lacks diversity in patient demographics	Regional activity changes are significant for pain, but sample size limits broader applicability
Michels et al. (2011) [83]	23	57	Chronic neuropathic pain	Eyes closed	Theta, alpha, beta, gamma	Increased power in theta and alpha; higher power in high pain relief group	Notable increases in fronto-central and temporo-parietal regions	Control group comparison, large sample size	Lack of information on pain intensity variations	Clear patterns of spectral changes linked to pain relief, though further studies needed for causal analysis
Vuckovic et al. (2018) [84]	31	45	Subacute SCI with NP	Eyes open/closed	Theta, alpha, beta	Reduced reactivity in NP groups, higher gamma power in specific regions	Significant reactivity differences between groups, strong correlation with pain levels	Comprehensive analysis with multiple groups	No longitudinal data	Useful for predictive analysis of NP but needs larger sample size for conclusive results
Wydenkeller et al. (2009) [85]	26	47	SCI + chronic NP	Eyes closed	Theta, alpha	Slower EEG peak frequency in SCI pain group	Slow frequency in frontal regions	Clear EEG analysis method	Small sample size limits generalizability	Frequency shifts are significant but study lacks power for large-scale conclusions
Di Pietro et al. (2018) [86]	20	50.1	Chronic orofacial NP	Eyes closed	Theta, alpha, beta	Higher power in theta and alpha, no significant beta differences	Frontal and parietal cortex involvement in increased power	Small sample size, no control for medication effects	No significant pain intensity correlation	Power differences in theta and alpha suggest neurophysiological changes but require larger validation
Zhou et al. (2018) [87]	14	64.4	Post-herpetic neuralgia	Eyes closed	Gamma	Increased gamma activity in parietal and temporal regions	Increased gamma in right parietal/temporal and left prefrontal	Small sample size, gamma focus	Lack of broader pain spectrum considered	Gamma increase may be specific to PHN, further studies needed for generalization
Krupina et al. (2020) [88]	24	36.6	Multiple sclerosis + CNP	Eyes closed	Delta, theta, alpha, beta	Increased delta and theta power in MS with CNP, higher beta in prefrontal regions	Temporal and occipital beta increase in MS with CNP	Focused on MS patients with pain	No comparison with healthy control group	Key frequency increases may link to MS pathology, but broader comparisons needed
Levitt et al. (2020) [89]	20	54.25	Lumbar radiculopathy	Eyes open/closed	Theta, alpha, beta, gamma	Significant PSD differences in beta and low gamma in radiculopathy	Significant beta differences in C3 and CZ	Large sample, machine learning analysis	Lack of clear control for confounding factors like medication	Novel machine learning approach provides robust insights, but confounding variables could affect reliability
Teixeira et al. (2021) [90]	12	54.4	Chronic NP	Eyes open/closed	Beta	Lower beta power in chronic pain patients	Lower global beta power in both low- and high-beta sub-bands	Small sample size, no control over other treatments	Generalizability limited by small sample and non-randomized design	Beta power reduction is notable, but further research with larger cohorts is necessary
Meneses et al. (2016) [91]	21	47.92	Rheumatoid arthritis + chronic pain	Eyes closed	Theta, alpha	Increased alpha and theta power in chronic pain group	Alpha increase in parietal and occipital regions	Comparison with HC group	No control for disease severity	Enhanced alpha activity may reflect chronic pain but needs further exploration
Zolezzi et al. (2023) [92]	36	44	Neuropathic pain	Eyes open/closed	Delta, theta, alpha, beta, gamma	Increased delta, decreased theta and alpha, increased beta with eyes open	Changes in beta and gamma with eyes open/closed	Comprehensive frequency range analysis	Cross-sectional study, lacks longitudinal follow-up	EEG changes in various bands are evident but require larger, repeated measures studies
Wang et al. (2024) [93]	18	48.06	Spinal cord injury + NP	Eyes open/closed	Theta, alpha, beta	Higher PSD in β band for pain group	PSD differences in frontal and motor regions	Good electrode distribution, multiple conditions	Small sample size, no differentiation by pain intensity	Reveals power spectral shifts linked to pain but needs more participants for stronger conclusions
Rajan et al. (2024) [94]	26	48.5	Chronic neuropathic pain	Eyes closed	Delta, theta, alpha, beta	Higher delta and theta power in pain group; no significant alpha differences	Increased theta across all montages except Fp1 and F3	Larger sample size	No control for medication effects	Increased delta and theta power in pain patients suggests significant changes in brain activity, but potential confounding effects limit conclusions
van den Broeke et al. (2013) [95]	19	52	Chronic NP (breast cancer)	Eyes closed	Alpha	Greater alpha amplitude in pain patients	Enhanced alpha power across the scalp	Small sample, no control for tumor stages or other treatments	Limited sample size and lack of further controls may impact reliability	Alpha increase in breast cancer-related pain is significant but needs validation in broader populations

As a result of reviewing available EEG studies in patients with neuropathic pain, both common and divergent patterns of brain activity were observed. In most studies, patients with neuropathic pain exhibited increased EEG power in the lower frequency bands, particularly in the theta (4–9 Hz) and alpha (7–12 Hz) bands, suggesting enhanced neuronal synchronization. In some studies, such as those involving spinal cord injury [80] and patients with chronic neuropathic pain [83], a shift in the dominant EEG frequency towards lower values was noted. The dominant frequency was also lower in neuropathic pain patients compared to healthy controls [84, 93]. For patients with different types of neuropathies, including cancer-related neuropathic pain [95], pain associated with spinal cord injury [81], and post-herpetic neuralgia [87], the most prominent changes were observed in the alpha and beta bands. Increased alpha activity was particularly evident in patients with pain following breast cancer treatment [95], and in studies involving chronic neuropathic pain related to multiple sclerosis [88] and lumbar radiculopathy [89], increased power in the beta and gamma bands was also noted. Differences in EEG reactivity, particularly in the alpha and beta bands, suggest variability in brain response to various forms of neuropathic pain. Despite these common observations, the results differ in terms of the intensity and localization of changes in EEG. For instance, studies in patients with chronic neuropathic pain due to spinal cord injury [80] showed a significant reduction in brain reactivity to eye opening, particularly in the theta and alpha bands. In contrast, in studies of post-herpetic neuralgia [87], the predominant feature was increased gamma activity in the parietal and temporal regions. The variability in results may also stem from the methodologies used and electrode placements, as evidenced by different results for the same frequency bands (e.g., varying locations within the theta and alpha bands across studies). In conclusion, EEG findings in neuropathic pain demonstrate increased neuronal synchronization in the lower frequency bands, especially theta and alpha, as well as changes in beta and gamma activity that vary depending on the type and location of pain. While common patterns are observed across studies, the differences in results indicate the need for further consideration of variables such as electrode location and the specific characteristics of the study population, which may impact the interpretation of results and the application of these findings in clinical practice.

### Discussion on research examining EEG in neuropathic pain

NP is a common subject of neurophysiological studies involving EEG. We found 18 publications examining EEG in people with various types of NP. These studies are characterized by significant heterogeneity. Different types of NP were studied; the methodology of EEG measurements varied (different electrode montages, frequency band definitions, eyes open and closed conditions, relative and absolute power measurements); and the patients were of different ages (although mainly middle-aged and elderly). This heterogeneity is the reason for the discrepancies in the findings, but the studies repeat a common pattern of EEG activity typical of NP—an increase in theta activity and a decrease in alpha activity. Potential mechanisms of the EEG patterns in NP are discussed below.

#### Increased theta activity in NP

The first quite common element from EEG studies on NP is excess theta activity. This increase in theta power has been widely linked to the phenomenon of thalamocortical dysrhythmia (TCD), which plays a central role in the neurophysiological basis of chronic pain [96]. TCD proposes a sequential set of events that contribute to the development and maintenance of NP. The basic mechanism of TCD is the same for all sites where patients experience neurogenic pain [97]. First, a lesion, whether peripheral or central, leads to the deafferentation of excitatory inputs on thalamic relay cells [96]. This deafferentation initiates the neurogenic pain syndrome and results in disfacilitation and hyperpolarization of thalamic relay neurons [98]. The hyperpolarized state of these neurons triggers bursts of activity at theta frequency due to the deinactivation of calcium T-channels [98]. These bursting thalamic relay neurons exert a rhythmic influence on thalamocortical loops in the theta frequency band. The thalamus and cortex are densely and reciprocally interconnected, and their tight functional coupling is supported by various projections, including thalamocorticothalamic, thalamoreticulothalamic, and corticoreticulothalamic connections. This functional coupling between the thalamus and cortex is confirmed by high theta coherence between these two regions [99]. The tendency of the thalamocortical network to maintain a given functional modality reinforces the hyperpolarized state of thalamic relay neurons over time. This leads to the diffusion of low-frequency theta activity to an increasing number of neighboring thalamocortical loops, resulting in the widespread overproduction of slow rhythms in the awake brain, which characterizes TCD [96]. The excess theta power becomes measurable in thalamic local field potentials, magnetoencephalography (MEG), and of course electroencephalography recordings of patients with NP. The presence of bursts of theta activity, along with cortical determinants of the EEG such as refractory periods and axonal transmission latencies, contributes to the spread of excess EEG oscillations over the entire theta band. Furthermore, the dysregulated thalamocortical rhythm in NP could be associated with changes in cortical inhibitory mechanisms. Thalamocortical modules in theta mode exert less collateral inhibition on neighboring modules, leading to their overactivation in high-frequency beta ranges. This phenomenon, known as the “edge effect,” provides a ring of reduced inhibition onto the cortex surrounding the low-frequency theta area [100]. It has been hypothesized that aberrant thalamic neuron firing that occurs internally could interfere with thalamocortical networks and cause aberrant pain processing [101]. TCD also occurs in other pathologies, such as Parkinson’s disease, depression, and tinnitus [102]. The patterns of EEG activity in these disorders are similar, but their spatial distribution partly differs. The authors of the same study [102] utilized support vector machine (SVM) learning to analyze resting-state EEG oscillatory patterns in patients with Parkinson’s disease, neuropathic pain, tinnitus, and depression. The SVM model for pain achieved an accuracy rate of 92.53% (sd = 1.59) in distinguishing between pain patients and healthy controls, significantly outperforming a random model, which had an accuracy of only 52.74%.

Various forms of NP, such as post-stroke pain (thalamic pain syndrome) or peripheral neuropathic pain (e.g. in diabetes), may be associated with TCD. In thalamic pain, damage to the thalamus leads to permanent changes in its activity, which may result in chronic pain. In peripheral neuropathic pain, loss of sensory signals from the periphery may lead to similar changes in the activity of the thalamus and cerebral cortex [99]. In the case of diabetic neuropathy, studies have shown reduced functional connectivity between the thalamus and the cerebral cortex in patients with chronic painful diabetic polyneuropathy. In particular, reduced synchrony was observed between the primary somatosensory cortex (S1) and some thalamic nuclei involved in pain processing. Reduced connectivity between the ventrolateral posterior nucleus (VPL) and sensorimotor, frontal, cingulate, and parietal areas and between the medial dorsal nucleus (MDN) and sensory and emotional processing areas may reflect changes in pain processing [103]. Functional magnetic resonance imaging (fMRI) studies have shown that patients with chronic low back pain have abnormal functional connectivity between thalamic nuclei and the somatosensory cortex and insula. These changes are consistent with the presence of TCD and may reflect mechanisms of pain maintenance in cLBP [104].

Further exploration reveals more specific links between TCD patterns and clinical manifestations across different NP types. For instance, in trigeminal or orofacial neuropathic pain, TCD, often characterized by increased theta and low alpha power, is associated with altered thalamic firing and may contribute to clinical outcomes like increased pain sensitivity and potentially allodynia (pain from non-noxious stimuli). This may be related to reduced thalamic GABA content observed in these conditions [86, 105, 106]. While diabetic neuropathy shows altered thalamocortical functional connectivity consistent with TCD concepts [103], post-herpetic neuralgia (PHN) presents a TCD profile potentially marked by increased gamma activity, which positively correlates with clinical reports of pain intensity, anxiety, and depression [87]. In central pain syndromes like central post-stroke pain (CPSP) and spinal cord injury (SCI) pain, TCD is strongly implicated due to the common underlying thalamic lesions or dysfunction. Although direct correlations between TCD markers and specific CPSP symptoms like burning pain or thermal hypersensitivity require more research, thalamic changes (e.g., altered anatomy, reduced GABA) are evident [107–109]. Furthermore, surgical interventions targeting thalamic structures, thought to disrupt TCD, have shown efficacy particularly for intermittent pain and allodynia in some central pain patients [99]. In SCI pain, frontal theta power has been directly correlated with reported pain intensity [110], and reduced thalamic GABA is specifically found in SCI patients with neuropathic pain compared to those without, reinforcing the link between thalamic dysfunction, TCD, and the clinical pain experience [111]. Beyond sensory symptoms, TCD in chronic pain has also been linked to broader clinical outcomes such as disturbances in motor performance and cognition [112]. These diverse findings underscore that while TCD provides a fundamental framework, its specific electrophysiological signature (e.g., dominant frequencies, connectivity patterns) and clinical correlates can vary depending on the etiology and characteristics of the neuropathic pain condition. Recognizing the involvement of TCD in various types of pain opens up new therapeutic possibilities, including through the use of neuromodulation (e.g. deep brain stimulation) or neurofeedback techniques.

#### Decrease in alpha activity in NP

Across various studies examining EEG patterns in patients with NP, general trends in alpha activity in patients with NP reveal several consistent patterns. Multiple studies report a reduction in absolute and relative alpha power in patients with NP. This reduction is particularly notable in individuals with spinal cord injuries, multiple sclerosis with central neuropathic pain, and chronic neurogenic pain. Next consistent finding across various studies is the shift of dominant alpha frequency towards lower frequencies in patients with NP. This shift has been observed in diverse populations, including those with spinal cord injuries and chronic lumbar radiculopathy. Regional variations in alpha activity are also noted, with higher power often found in posterior regions (parietal and occipital lobes) and reduced power in frontal regions, indicating altered cortical activity patterns associated with chronic pain conditions. Additionally, patients with NP generally exhibit reduced reactivity in alpha band power when transitioning from eyes closed to eyes open conditions, suggesting a less adaptive neural response to sensory input changes.

The reduction in alpha power is often more pronounced in certain brain regions. For instance, significant reductions in alpha activity are frequently observed in frontal regions. There is a strong correlation between increased activity in the thalamus and alpha activity in the cortex, indicating that the thalamus is important for the production of alpha rhythms [113, 114]. Lesion investigations that showed thalamic lesions resulted in aberrant or abolished alpha rhythms in the cortex corroborated this theory [115]. It has been shown from the standpoint of functional imaging that NP differs from non-neuropathic pain in key ways and can be caused by anatomical alterations in the thalamus [116, 117]. There is mounting evidence that the thalamus plays a crucial role in the production and/or persistence of NP. For instance, NP has been linked to thalamocortical dysrhythmia [98], changed thalamic architecture [118], modifications in thalamic biochemistry and decreased thalamic perfusion [100], which together may result in decreased cortical alpha activity in NP.

It is thought that gamma-aminobutyric acid (GABA) inhibition produces the cortical alpha rhythm [119–121]. Numerous investigations have demonstrated a decreased GABAergic inhibitory activity in conjunction with NP [122, 123]. Moreover, neuronal hyperexcitability, which results in mechanical allodynia and NP, is mostly dependent on astrocyte GABA-mediated tonic excitation [124]. This may result in a reduction of alpha activity in NP. Overall, alpha activity levels in relation to NP also may be a reflection of cortical inhibition or activation, and alpha rhythms linked to the processing of painful stimuli may also fall under the functional inhibition theory. Moreover, the global reduction of spontaneous alpha oscillatory activity brought on by the painful stimuli is consistent with extensive brain activation and a change in the perception of pain [125]. Additionally, alpha activity in the brain is known to play a role in attentional processes. It is believed that alpha waves help in inhibiting irrelevant sensory information, allowing individuals to focus their attention on specific stimuli. In the context of NP, the reduced alpha activity may result in an impaired ability to shift attention away from pain-related stimuli. This heightened attention to pain can contribute to increased pain vigilance, rumination, and magnification, which are commonly observed in chronic pain conditions [126].

## Results of studies on neurofeedback

**Fig. 2:**
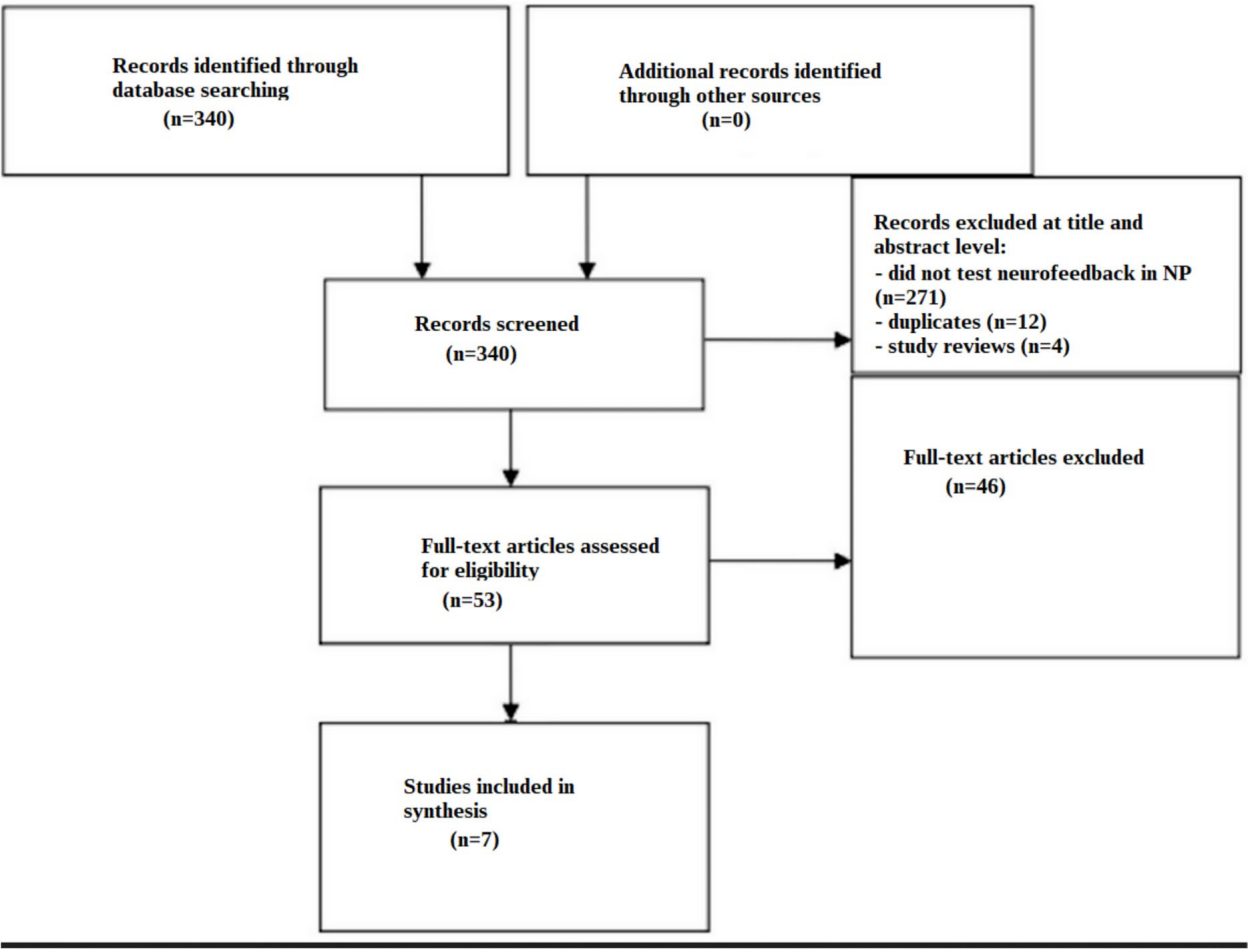
Second flow chart depicting the different phases of the review

The second screening process is depicted in a flow chart (Fig. 2). Initially, utilizing search techniques across many databases, 340 studies were found. After examining the titles and abstracts of these studies, 287 were disqualified; 271 of them did not test neurofeedback in NP, 12 of them were duplicates, and four of them were research reviews. The final 53 studies were then subjected to a thorough full-text evaluation. Upon careful examination of the texts, it was determined that seven articles satisfied the requirements for inclusion. The neurofeedback studies included in this review [127–133] were published between 2015 and 2022. A total of 107 participants took part in all studies. Among the included studies, three were pilot studies [127, 129, 133], one was a usability study [132], and three [128, 130, 131] did not specify the type of study. Two studies [130, 131] included a control group. Patients included in the studies were on average 50 years old.

### Studies examining neurofeedback in the treatment of neuropathic pain—an overview

Neurofeedback research in NP is summarized in Table 3. The study conducted by Hassan et al. [127] aimed to explore how neurofeedback training affects central neuropathic pain (CNP) and its underlying brain patterns in individuals with chronic paraplegia. Seven patients (age 50 ± 4) participated in the study. The training consisted of two phases: audio neurofeedback and visual neurofeedback. During the audio neurofeedback phase, patients listened to feedback provided from the occipital region with their eyes closed, aiming to induce relaxation. The training parameters were set in the lower α band (7–10 Hz) at the occipital region to accommodate the lower peak frequency observed in patients with CNP. The relative power was calculated within the 2–30 Hz frequency range. The goal of the training was to increase α band power while reducing θ (4–8 Hz) and β (12–30 Hz) band powers. Specifically, the sensory motor rhythm (SMR) in the β band (12–15 Hz) was targeted. Electrodes placed over the primary motor and sensory cortex (C4/C3/Cz/P4) were used one at a time for the training. Four protocols were tested: Protocol 1 rewarded SMR and inhibited θ and β at Cz, Protocol 2 rewarded α and inhibited θ and β at P4, Protocol 3 rewarded α and inhibited θ and β at C3, and Protocol 4 rewarded α and inhibited θ and β at C4. Out of the seven patients with pain who participated in the study, five completed it. Patients reported varying levels of pain reduction during the different protocols. Protocol 1 did not result in any pain reduction, while Protocol 2 led to a moderate reduction for patients P2 and P4. Protocols 3 and 4 showed significant pain reduction, with pain levels dropping to 0–2 on the visual numerical scale during the training. However, patients using Protocol 3 experienced strong spasms characterized by uncontrollable movements of their paralyzed legs while practicing neurofeedback. As a result, Protocol 4 was used for most of the training days as it provided the best pain relief with minimal side effects. Six out of seven patients reported immediate pain reduction during the second or third daily treatment with Protocol 4. Although initially short-term during the neurofeedback sessions, all five patients who received 20 or more treatment sessions achieved a statistically significant and clinically significant (> 30% reduction from the individual’s initial pain intensity) reduction in pain. The long-term pain reduction persisted for several weeks after the completion of the therapy. One month after the treatment, patients reported reduced pain intensity, although it had increased by 1–2 grades on the visual numerical scale compared to the last neurofeedback session.

**Table 3:** Neurofeedback studies of neuropathic pain included in this review

Study reference	Objective	Participants (number and condition)	Neurofeedback protocols	Electrode placements	Frequency bands targeted	Number of sessions	Outcomes (pain reduction)	Additional observations/findings
Hassan et al. (2015) [127]	To explore how neurofeedback training affects CNP and brain patterns in individuals with chronic paraplegia	7 patients with chronic paraplegia	Protocol 1: Reward SMR, inhibit θ and β at Cz; Protocol 2: Reward α, inhibit θ and β at P4; Protocol 3: Reward α, inhibit θ and β at C3; Protocol 4: Reward α, inhibit θ and β at C4	Primary motor and sensory cortex (C4/C3/Cz/P4)	α (7–10 Hz), θ (4–8 Hz), β (12–30 Hz)	20 + sessions	Significant pain reduction for most patients, especially with Protocol 4	Protocol 4 provided the best pain relief with minimal side effects; long-term pain reduction persisted for weeks
Hasan et al. (2021) [128]	To understand how neurofeedback impacts deeper cortical structures in the “pain matrix”	5 paraplegic patients with CNP	Decrease θ (4–8 Hz) and β (20–30 Hz); increase α (9–12 Hz)	Sensory-motor cortex (C4, Cz, C3, P4, Oz)	α (9–12 Hz), θ (4–8 Hz), β (20–30 Hz)	Up to 40 sessions	Reduction in pain on VAS for each patient	Significant pain reduction observed with consistent neurofeedback training
Vučković et al. (2019) [129]	To investigate self-managed neurofeedback treatment on CNP in individuals with chronic spinal cord injury	15 participants with chronic CNP	Upregulate α (9–12 Hz), downregulate θ (4–8 Hz) and β (20–30 Hz)	C4	α (9–12 Hz), θ (4–8 Hz), β (20–30 Hz)	Not specified	Significant pain reduction in 8 out of 12 participants	Larger increase in α power and decrease in θ power when targeting individualized frequency bands
Anil et al. (2022) [130]	To explore the relationship between neurofeedback learning and the use of mental strategies	25 non-CNP participants, 10 CNP participants	Reinforce brain activity in 9–12 Hz, suppress 4–8 Hz and 20–30 Hz	C4	α (9–12 Hz), θ (4–8 Hz), β (20–30 Hz)	4 sessions	No specific mental strategy strongly associated with neurofeedback success	Successful non-CNP participants used imagination-related strategies more frequently
Hasan et al. (2016) [131]	To investigate the relationship between pain intensity reduction and activation changes in the sensory-motor cortex during imagined movements	25 volunteers (5 CNP, 10 non-CNP paraplegic, 10 able-bodied)	Increase α (9–12 Hz), decrease θ (4–8 Hz) and β (20–30 Hz)	Primary motor cortex (C4, C3) and parietal region (P4)	α (9–12 Hz), θ (4–8 Hz), β (20–30 Hz)	Not specified	Reduced α ERD and β ERD in patients with pain during motor imagination	ERD became more confined to specific frequency bands post-neurofeedback
Al-Taleb et al. (2019) [132]	To assess the usability and effectiveness of a neurofeedback system for home-based treatment of CNP	15 participants with spinal cord injury and CNP	Raise dominant α frequency, reduce θ and β bands	Between C4 and C2	α (9–12 Hz), θ (4–8 Hz), β (20–30 Hz)	2 months of neurofeedback	No significant correlations found between pain level and various factors	High usability and satisfaction with the neurofeedback system
Hasan et al. (2019) [133]	To confirm the effectiveness of a specific neurofeedback training method for managing CNP	5 participants with spinal cord injury and CNP	Reward α, inhibit θ and β at C4	C4	α (9–12 Hz), θ (4–8 Hz), β (20–30 Hz)	Various, up to 34 sessions	Statistically significant pain reduction in all patients	Protocol 4 found to be the most effective

The study conducted by Hasan et al. [128] aimed to understand how neurofeedback training impacts the deeper cortical structures involved in processing neuropsychological functions, specifically within the “pain matrix.” The research involved five paraplegic patients (age 51 ± 3) who experienced CNP. These patients underwent a series of up to 40 neurofeedback training sessions. During the training, the researchers measured EEG relative power in the sensory-motor cortex, specifically at the C4 site. The participants were instructed to decrease the power within the theta (4–8 Hz) and beta (20–30 Hz) frequency bands while simultaneously increasing the power within the alpha (9–12 Hz) frequency band. The threshold for success was set at 10% below or above the baseline value recorded during the initial training session. Each participant received up to 40 neurofeedback training sessions, with a frequency of three sessions per week. In most training sessions, five electrodes were placed on specific locations on the head (Cz, C3, C4, P4, Oz). However, on one training day, 16 electrodes were used. Each patient reported a reduction in pain on the visual analog scale (VAS) on the day of training. The baseline neurofeedback session was compared to the subsequent neurofeedback session (DNF), with the pain scores showing a decrease for each patient: P1 (6/5), P2 (6/5), P3 (5/3), P4 (7/4), and P5 (9/6).

In the feasibility study conducted by Vučković et al., [129] the authors investigated the effects of self-managed neurofeedback treatment on CNP in individuals with chronic spinal cord injury. The study included 15 participants (aged 50.6 ± 14.1) with chronic CNP, and they underwent initial training in the hospital before practicing neurofeedback at home. During the neurofeedback sessions, the participants were instructed to upregulate the alpha (9–12 Hz) and downregulate the theta (4–8 Hz) and higher beta (20–30 Hz) frequency bands. The neurofeedback protocol involved electrode placement at location C4, and the sessions lasted for 30 min. The researchers collected data on pain levels before and after neurofeedback, as well as EEG recordings before and during neurofeedback. Pain questionnaires were also utilized. The analysis of the EEG data revealed that most participants successfully regulated their alpha band activity during neurofeedback. However, it is worth noting that the individual alpha band frequencies were lower in the participants compared to the general population, with an average dominant frequency of αp = 7.6 ± 0.8 Hz (median 8 Hz) compared to the average of around 10 Hz in the general population. Out of the 15 participants, ten significantly increased their individual alpha power (αp ± 2 Hz) during neurofeedback, while four participants increased the power in the fixed alpha band (8–12 Hz). Eight out of the 12 participants who experienced a significant reduction in pain also significantly upregulated their individual alpha band power. Furthermore, the study found that there was a significantly larger increase in alpha power and a decrease in theta power when participants targeted their specific individualized frequency bands rather than fixed frequency bands.

Anil et al. [130] conducted a study to explore the relationship between neurofeedback learning and the use of mental strategies in participants. The study included 25 participants without a clinical CNP condition and ten participants with CNP (mean age = 51.70, SD = 10.55). All participants underwent neurofeedback training on four separate visits, where they were given instructions to reinforce brain activity in the 9–12 Hz frequency range and suppress activity in the 4–8 Hz and 20–30 Hz ranges at the C4 site. After each visit, participants were interviewed about the mental strategies they employed. Questionnaires were also used to assess factors like self-efficacy, locus of control, motivation, and workload related to the neurofeedback training. The mental strategies reported by participants were categorized into two groups: mental strategies involving goal-directed mental actions and affect, which referred to the emotional experiences during neurofeedback. The findings showed that successful non-CNP participants utilized imagination-related mental strategies more frequently and reported experiencing more negative emotions compared to successful CNP participants. However, no specific mental strategy was found to be strongly associated with neurofeedback success. There was some indication that the lack of success was linked to negative emotions. Self-efficacy showed a moderate correlation with neurofeedback success (*r* = < 0.587, *p* = < 0.020), while locus of control, motivation, and workload had weak and non-significant correlations (*r* < 0.300, *p* > 0.05).

The study of Hasan et al. [131] aimed to investigate the relationship between pain intensity reduction and activation changes in the sensory-motor cortex during imagined movements. The study involved 25 volunteers divided into three groups: Group 1 consisted of five paraplegic patients with CNP (age 50 ± 4), group 2 included ten paraplegic patients without CNP, and group 3 comprised ten able-bodied individuals with no chronic pain (AB). The study employed a cue-based motor imagination protocol, where participants were instructed to imagine specific movements while their brain activity was recorded using EEG. The purpose of motor imagination was to induce activity in the cortico-spinal tract, and EEG responses were measured to assess the modulation of motor cortex activity. Neurofeedback training sessions were conducted for the patients in group 1, and the training technique involved operant conditioning based on non-verbalized rules. The patients were trained to increase the relative power in the alpha (9–12 Hz) frequency band while simultaneously decreasing the power in the theta (4–8 Hz) and higher beta (20–30 Hz) frequency bands. Neurofeedback was provided from electrode locations over the primary motor cortex (C4, C3) and the parietal region (P4). The results showed that following neurofeedback training, there were changes in event-related desynchronization (ERD) and event-related synchronization (ERS) patterns during motor imagination tasks. Specifically, reductions in alpha ERD and beta ERD were observed in the patients with pain (group 1), suggesting reduced deactivation during motor imagination. The reduction in beta ERD was not related to baseline power changes and was more likely indicative of decreased deactivation during motor imagery. Moreover, the ERS/ERD maps over the electrode locations Cz and C3 showed that after neurofeedback, the ERD became more confined to specific frequency bands (alpha and beta bands) in the patients with pain. This shift resembled the typical response observed in able-bodied individuals without pain. Additionally, reductions in ERD intensity were observed in the patients with pain during motor imagination tasks involving their unaffected upper limbs (group 1).

The study of Al-Taleb et al. [132] aimed to assess the usability and effectiveness of a neurofeedback system for home-based treatment of CNP in individuals with spinal cord injury. The study used a combination of usability testing methods, including usability testing by end users, analysis of recorded data (EEG, electronic pain diary), inquiry methods (observation, interviews, questionnaires), and functional testing of the neurofeedback software application on able-bodied participants. The study employed both subjective and objective measures of usability. Subjective measures included interviews, questionnaires, and pain rating, while objective measures included EEG measurement. Three validated questionnaires were used to assess pain location and level (Brief Pain Inventory), symptoms of NP (Neuropathic Pain Symptom Inventory), and satisfaction with the system usage (Quebec User Evaluation of Satisfaction questionnaire). Additionally, custom-made questionnaires were used to assess participants’ attitudes towards using new technology, previous experience with non-pharmacological treatments of CNP, and their experience with practicing neurofeedback. The electrode placement for neurofeedback training was positioned towards the back, behind an imaginary vertical line drawn through the patients’ ears. It was positioned approximately between the C4 and C2 electrode locations. The decision to focus on a slightly higher alpha band (9–12 Hz), excluding the lowest frequency (8 Hz), was based on the observation that individuals with spinal cord injury and CNP tend to have a lower dominant alpha frequency compared to both able-bodied individuals and those with SCI but no pain. The aim was to raise the dominant alpha frequency through neurofeedback training and enhance the power of the alpha band. Simultaneously, participants were also instructed to reduce the power of the theta and higher beta bands. Out of the initially recruited 20 participants (age 50.6 ± 14.1), 15 decided to participate in the home-based NFB study. Eight participants discontinued the study due to various reasons, while seven completed the required 2 months of neurofeedback. EEG data and pain diaries were collected from all 15 participants, and nine participants took part in final interviews and filled out user experience questionnaires. The study found no significant correlations between pain level and injury level, pain level and time since injury, pain level and reduction of pain during neurofeedback, dominant alpha frequency and initial pain level, and dominant alpha frequency and reduction in pain during neurofeedback. Additionally, there was no significant difference in initial pain level between walkers and non-walkers or between participants with incomplete and complete injuries.

The purpose of the research paper by Hasan et al. [133] was to confirm the effectiveness of a specific neurofeedback training method for managing CNP in patients with spinal cord injuries. The study examines the impact of baseline measurements on the training protocol. Feedback was given from the C4 location, with inhibiting theta and beta waves and rewarding alpha waves. Two types of baselines were used: Baseline 1, recorded on the first day of training and used as a common baseline throughout, and Baseline 2, which involved recording a different baseline each day before starting the training in the pre-neurofeedback state. A total of five participants took part in the study. Four different protocols were tested, and Protocol 4, which involved rewarding alpha waves and inhibiting theta and beta waves at the C4 site, was found to be the most effective in reducing pain through modulation of EEG activity. Patient 1 (P1) received only nine sessions with Protocol 4 because they had undergone training with other protocols in the initial days. Patient 2 (P2) received 34 sessions, while Patient 3 (P3) and Patient 4 (P4) received 27 sessions each with Protocol 4. Patient 5 (P5) received 18 sessions of Protocol 4 training, as this patient was hospitalized for 3 weeks specifically for neurofeedback training. All five patients experienced statistically significant pain reduction.

### Discussion on studies examining neurofeedback in the treatment of neuropathic pain

Based on the study overview examining neurofeedback in the treatment of NP, several similar neurofeedback protocols were employed. Most studies focused on upregulating the alpha frequency band (typically 9–12 Hz) and downregulating the theta (4–8 Hz) and higher beta (20–30 Hz) frequency bands. Common electrode placements included sites such as C4, Cz, P4, and occipital regions. Specific protocols often used Cz (central), C3/C4 (sensory-motor cortex), and P4 (parietal) locations. Several studies used multiple protocols to determine the most effective training parameters. For example, different protocols were tested by rewarding specific frequency bands while inhibiting others at various electrode locations. Protocols included variations like rewarding sensory motor rhythm (SMR) in the beta band (12–15 Hz) while inhibiting theta and higher beta at Cz, rewarding alpha and inhibiting theta and beta at P4, C3, and C4.

Training often included both audio and visual feedback modalities. Audio feedback was sometimes used initially to induce relaxation, with visual feedback providing real-time brain activity monitoring during training sessions. The sessions typically lasted around 30 min and were conducted multiple times per week, with the number of sessions varying from study to study. For example, participants received up to 40 sessions, with a frequency of three sessions per week. Some studies included home-based neurofeedback training, where participants practiced neurofeedback at home after initial training in a clinical setting. This approach aimed to assess the feasibility and effectiveness of self-managed neurofeedback. Adjustments were made to the frequency bands to match individual participants’ dominant frequencies, which were often lower in individuals with chronic pain compared to the general population. This individualized approach aimed to enhance the effectiveness of the training. Studies exploring the relationship between neurofeedback success and mental strategies found that participants used various mental strategies, including goal-directed mental actions and affective experiences. Pain reduction was measured using visual numerical scales or visual analog scales, with significant reductions reported in most studies after neurofeedback training. EEG relative power changes were also monitored to assess the effectiveness of the neurofeedback in modulating the targeted frequency bands. Neurofeedback induced EEG changes that were associated with symptom improvement. In study [127], EEG results during neurofeedback showed a widespread modulation of power across all three frequency bands, with the most significant changes in coherence observed in the beta band. The standardized low-resolution electromagnetic tomography analysis of EEG taken before and after neurofeedback therapy indicated a statistically significant reduction in beta band power in all patients. The areas exhibiting reduced power included the dorsolateral prefrontal cortex, the anterior cingulate cortex, and the insular cortex. In study [128], the alpha and beta band activity saw the most significant increase in the insular, cingulate, and frontal cortex regions, as well as in areas related to executive and emotional function processing.

Conversely, theta band activity decreased in the frontal, cingulate, and motor cortices. When analyzing the group as a whole, there was a notable reduction in both theta and beta band activity. In study [129], the alpha frequency band was the most successfully regulated during neurofeedback. Ten out of 15 participants significantly increased their individual alpha power.

Of the 12 participants who experienced a significant reduction in pain, eight showed a significant increase in their individual alpha band power. In study [131], there was a significant reduction in the alpha and beta bands, with the largest reduction occurring in the theta band.

Consequently, the cortical activity of the participants became similar to that of the two other groups without pain. In the study [132], participants successfully upregulated their alpha band power. In particular, they upregulated their individual alpha peak frequency.

A discussion of neurofeedback mechanisms in NP and future research directions are presented in Sect. 5 and 7.

## Design of neurofeedback protocols based on EEG evidence and discussion of potential mechanisms of action

There are increasing reports of the possibility of using neurofeedback protocols in patients with NP. The published results are positive in this regard, but special attention should be paid to the limitations of the mentioned studies, such as lack of control groups or placebo conditions, small sample sizes, and inconsistent protocols. Below we present preliminary proposals based on the reviewed EEG evidence, which, however, due to the mentioned limitations, require validation through rigorous research.

### Theta-focused neurofeedback protocol proposal

Based on the consistent finding of increased theta activity in NP, a potential neurofeedback protocol could focus on normalizing this rhythm as summarized in Table 4.

**Table 4:** Proposed theta-lowering neurofeedback protocol

Component	Details	Rationale/evidence
Primary objective	Reduce excessive theta power (4–7 Hz)	To normalize theta activity commonly elevated in NP [79, 81–83, 86, 88]
Secondary objectives	- Improve cortical synchronization - Normalize theta/beta ratio	Address potentially enhanced theta coupling [79]; Monitor theta/beta interactions
Target regions/electrode placement	- Posterior/parietal: P3, O1, O2 - Fronto-central: Fz, FCz, Cz - Frontal: FP1, FP2 - Temporal/periinsular: T3, T4, FC5	Based on regions showing increased theta activity [79, 81–83, 86, 88] or involvement in theta/alpha interactions [82]
Training approach	Provide feedback contingent on decreasing theta power in target regions. Monitor theta/beta ratio, potentially encouraging beta increases where appropriate [79]	Direct training to reduce the identified biomarker (excess theta). Balanced approach considering frequency interactions
Potential mechanisms (discussed below)	Modulation of thalamocortical dysrhythmia (TCD)	In Sect. 5.1.1

#### Potential mechanisms of theta-lowering neurofeedback

Mechanisms of theta-lowering neurofeedback can be elaborated based on the concept of thalamocortical dysrhythmia (TCD) and the role of the thalamus in NP. Neurofeedback training targeting theta reduction might:

- Normalize the resting membrane potential of hyperpolarized thalamic relay neurons, thereby decreasing the bursts of theta frequency activity [Related to TCD mechanism, 98].
- Reduce the deinactivation of calcium T-channels linked to the hyperpolarized state, resulting in less rhythmic theta influence on thalamocortical loops [Related to TCD mechanism, 98].
- Modulate thalamocortical functional coupling, potentially reducing the pathologically high theta coherence observed in NP patients by reinforcing normal communication patterns [97, 99].
- Prevent the spread of low-frequency theta rhythms to neighboring thalamocortical loops by decreasing overall thalamic theta activity [Related to TCD mechanism, 96].
- Enhance cortical inhibitory mechanisms that may be dysregulated in NP, helping restore proper collateral inhibition and reducing consequent high-frequency overactivation (edge effect) [100].
- Correct aberrant firing patterns of thalamic neurons, improving thalamocortical network function and reducing aberrant pain processing [101].
- Directly reduce the excess theta power measurable in EEG, leading to a more balanced brain rhythm.

### Alpha-focused neurofeedback protocol proposal

Given the common finding of reduced and slowed alpha activity in NP, protocols aimed at enhancing alpha power and normalizing its frequency could potentially alleviate pain, as summarized in Table 5

**Table 5:** Proposed alpha-enhancing neurofeedback protocol

Component	Details	Rationale/evidence
Primary objective	Increase alpha power (typically 8–12 Hz, but individualized)	To counteract reduced alpha activity commonly observed in NP and potentially leverage alpha’s role in sensory gating/inhibition
Secondary objectives	- Normalize dominant alpha frequency - Enhance alpha coherence - Improve alpha reactivity (eyes open vs. closed)	Address slowed alpha peak frequency [80, 134]; Normalize disrupted cortical communication [79, 81]; Improve adaptive neural responses
Target regions/electrode placement	- Fronto-central: FP1, FP2, F3, FZ, F4 - Parieto-occipital: PZ, P3, P4, O1, O2	Target regions showing significant alpha alterations or involvement in sensory processing/reactivity [81]
Training approach	Provide feedback contingent on increasing alpha power in target regions. Customize target frequency based on individual's dominant alpha frequency [80, 134]. Consider training alpha coherence between posterior and frontal regions [79, 81]. Tailor intensity based on NP profile/response [83]	Individualization may enhance effectiveness. Coherence training targets network communication. Customization adapts to patient variability (e.g., High vs. Low Pain Relief responders [83])
Potential mechanisms (discussed below)	Modulation of thalamocortical connectivity, GABAergic inhibition, pain processing pathways, and attentional control	In Sect. 5.2.1

#### Potential mechanisms of alpha-enhancing neurofeedback

Neurofeedback aimed at enhancing alpha activity in NP might work through several potential mechanisms:

- Modulating thalamocortical loops: Increasing thalamic-cortical connectivity might help restore normal alpha rhythms potentially disrupted by thalamic abnormalities associated with NP [113, 114, 117, 118]. It could also induce beneficial plastic changes in thalamic function.
- Enhancing GABAergic inhibition: Promoting alpha activity may enhance cortical GABAergic inhibitory activity, which is crucial for alpha generation [119–121] and often reduced in NP [122, 123]. This could counteract neuronal hyperexcitability [124].
- Inhibiting pain processing: Enhanced alpha rhythms might contribute to the inhibition of pain processing pathways in the cortex, potentially reducing pain perception by modulating the brain's response to noxious stimuli [125]. Increased spontaneous alpha could counter broad brain activation associated with pain [125].
- Improving attentional control: Boosting alpha activity could enhance the brain’s ability to inhibit irrelevant sensory information, improving focus away from pain [126]. This may help reduce pain vigilance, rumination, and magnification common in chronic pain [126].

### Caveats and future directions

These proposed protocols are based on current EEG findings in NP. However, the reports underpinning these suggestions appear promising but have several important limitations, including the lack of control groups or placebo conditions, small sample sizes, and inconsistent protocols. All of these limitations significantly underscore the need for rigorous, large, randomized controlled trials (RCTs) to confirm these findings and validate specific neurofeedback approaches before they can be considered standard clinical practice.

## Neurofeedback—a chance for older people with NP?

The brain is subject to natural aging processes, which are visible in the EEG—there is a significant decrease in the alpha activity amplitude (8–13 Hz), (ii) slowing of the background activity (dominant alpha rhythm), and (iii) global increase in the power of theta (4–8 Hz) and delta (1–4 Hz) [135]. Taking this into account, as well as the increase in the incidence of NP in older people, it can be assumed that changes in EEG caused by aging predispose to the occurrence of NP. Neurofeedback therefore seems to be an ideal option to reverse this tendency and to treat and prevent the occurrence of NP in older people. Neurofeedback has significant advantages over other therapeutic interventions in this group of patients. Firstly, it is non-invasive, safe, and does not cause major side effects. It can be used by people using other forms of treatment and neurofeedback will not interfere with them. This is particularly important in older patients undergoing polypharmacy, which, with the addition of another drug, causes intensification of side effects and deterioration of well-being. Secondly, NP treatment through neurofeedback is precise and can be tailored to the individual patient using normative modelling. By performing a quantitative EEG of a patient with NP, the most appropriate protocol can be designed to suit the patient’s unique EEG activity pattern. Third, the use of neurofeedback in NP may bring additional positive effects in older patients suffering, for example, from cognitive decline. The introduction mentioned that cognitive problems in older people result in poorer diagnosis of NP, which may result in late initiation of NP treatment and prolongation of the patient’s suffering. The appearance of scattered theta waves, which are represented in a widespread increase in theta strength, and the sporadic occurrence of temporally predominant delta waves are further characteristics of EEG in old age [136, 137]. A reduction in the posterior alpha rhythm [138, 139] and a rise in slow activity (delta and theta) [140, 141] are key EEG aspects that are seen to be exaggerated in older people with neurocognitive disorders. Furthermore, the worsening cognitive performance throughout aging has been associated with slow wave activity [142, 143]. Neurofeedback training has also been used to improve cognitive functions in older people, whereby theta activity is inhibited [144], sensorimotor rhythm (SMR) [145] and alpha activity [146] are enhanced. This convergence of protocols targeting NP and cognitive decline means that patients could have a double benefit through neurofeedback involving improved cognitive function.

## Research limitations and future research directions

### Research limitations

Despite the promising results presented in this review, several important limitations must be acknowledged, both within the scope of the analyzed literature and within the current study itself. First, the studies included in this review exhibit significant methodological variability, which may have influenced the reported outcomes. These variations include differences in EEG measurement protocols (e.g., electrode montages, frequency band definitions, eyes open vs. eyes closed conditions), sample sizes, and participant demographics. Such heterogeneity limits the ability to generalize the findings to different patient populations and neuropathic pain (NP) types. Moreover, many studies lacked appropriate control groups and did not account for confounding variables, such as medication use or comorbidities, which are known to influence EEG patterns. This introduces a degree of uncertainty regarding the causality of the observed EEG changes in response to NP. Furthermore, the small sample sizes in many studies complicate the interpretation of the results. Although promising trends in EEG activity, such as increased theta activity and decreased alpha activity in NP patients, were observed, the limited sample size reduces the statistical power of these studies. Therefore, the findings should be treated with caution, and more robust studies with larger sample sizes are necessary to confirm these observations.

Additionally, this review focused primarily on summarizing the reported findings and potential mechanisms, rather than providing an in-depth analysis and interpretation of the specific outcome measures used in the included neurofeedback studies. A more detailed discussion exploring the clinical significance versus statistical significance of the reported changes, the correlation between different types of outcomes (e.g., EEG modulation and pain relief), and the limitations of the chosen assessment tools would be beneficial but was beyond the primary scope of this mechanistic review.

There is also a need for further development and refinement of methodologies, including a more detailed analysis of various confounding factors, such as the impact of medication on EEG outcomes. In relation to the current review itself, it is important to note that while an extensive literature search was conducted, the inclusion of only studies published in English may have led to publication bias, excluding potentially relevant studies published in other languages. Additionally, while the review attempted to focus specifically on the application of neurofeedback for NP treatment, it could have benefitted from a more detailed exploration of the mechanisms through which neurofeedback influences neuroplasticity and pain modulation, especially in the elderly population, which remains underrepresented in the existing literature.

### Future research directions

Future studies should address the limitations discussed above, beginning with the standardization of EEG measurement methodologies. Standardizing electrode placements, frequency band definitions, and the inclusion of different states such as eyes open and closed would significantly improve the comparability of results across studies. Additionally, future research should include larger, more diverse patient populations and the use of control groups to provide more reliable assessments of the effects of neurofeedback on NP. Larger cohorts, including different age and ethnic groups, will help determine whether the results of neurofeedback are consistent across various populations, thereby increasing the generalizability of the findings. Long-term studies are also crucial to evaluate how EEG abnormalities in NP patients may evolve over time and how prolonged neurofeedback intervention influences pain symptoms. Longitudinal monitoring of changes in brain activity will provide valuable insights into the durability of therapeutic effects and the potential mechanisms by which neurofeedback leads to improvements in brain function in NP patients. These studies should also examine the relationship between the duration of neurofeedback training and the maintenance of symptom relief.

There is also a pressing need for a deeper understanding of the neurophysiological mechanisms behind neurofeedback in NP treatment. While this review identifies potential mechanisms such as thalamocortical dysrhythmia and neuroplasticity, future studies should aim to clarify how specific neurofeedback protocols can target these mechanisms to improve pain management outcomes. In particular, research should focus on which EEG frequencies and brain activity patterns are most effective in alleviating NP symptoms across different types of pain. Moreover, research should investigate the potential synergistic effects of neurofeedback when combined with other therapeutic modalities, such as pharmacological treatments or physical therapy. Special attention should be given to the elderly population, who often experience NP, as more complex and coordinated therapeutic approaches may be needed, involving both neurofeedback and other forms of treatment, such as medication or physical therapy. In the context of NP management in the elderly, it is essential to develop neurofeedback protocols that address the specific challenges of this patient group, such as age-related brain structural changes, multimorbidity, and polypharmacy. Research focused on adapting neurofeedback to meet the needs of elderly patients is critical, especially given the higher risk of drug interactions and complications related to NP treatment in this population. Understanding how neurofeedback can be tailored to the needs of older adults will be key to its widespread clinical application.

In conclusion, neurofeedback represents a promising therapeutic option for NP; however, its application is still in the early stages. Future research should focus on refining methodologies, increasing sample sizes, and exploring the mechanisms behind neurofeedback to enhance its efficacy and expand its applicability in the treatment of neuropathic pain, particularly in vulnerable populations such as the elderly. We know little about the mechanisms of action of neurofeedback in the treatment of NP. Future research using advanced neuroimaging tools such as fMRI, MEG, and PET is necessary. Understanding these mechanisms can inform the optimization of neurofeedback protocols and improve their effectiveness. It is also worth using other potentially useful blood biomarkers [147–151].

## Conclusions

In light of the presented findings, neurofeedback demonstrates potential as a promising approach for the treatment of neuropathic pain; however, it is crucial to emphasize that the current evidence remains preliminary and subject to significant limitations. While some studies suggest the possible efficacy of neurofeedback in alleviating pain symptoms, such as reductions in pain intensity and improvements in patients'quality of life, there is a lack of robust, high-quality evidence supporting its widespread effectiveness. Consequently, the assertion that neurofeedback has the potential to become a standard treatment modality is premature. Further research, particularly well-designed, randomized controlled trials with larger sample sizes, is required to provide more definitive evidence regarding its effectiveness in the management of neuropathic pain. Only upon the completion of such rigorous studies can neurofeedback be considered for broader clinical application.

## Data Availability

No new data were created or analyzed in this study. Data sharing is not applicable to this article.

## References

[1] Backonja MM. Defining neuropathic pain. Anesth Analg. 2003;97(3):785–90. 12933403 10.1213/01.ANE.0000062826.70846.8D

[2] Colloca L, Ludman T, Bouhassira D, Baron R, Dickenson AH, Yarnitsky D, et al. Neuropathic pain. Nat Rev Dis Primers. 2017;16(3):17002. 10.1038/nrdp.2017.2 PMC5371025 28205574

[3] Heitmann H, Biberacher V, Tiemann L, Gündisch D, Andlauer TFM, Mühlau M, et al. Prevalence of neuropathic pain in early multiple sclerosis. Mult Scler J. 2016;22(9):1224–30. 10.1177/1352458515613643 26480924

[4] Schreiber AK, Nones CF, Reis RC, Chichorro JG, Cunha JM. Diabetic neuropathic pain: physiopathology and treatment. World J Diabetes. 2015;6(3):432–44. 25897354 10.4239/wjd.v6.i3.432 PMC4398900

[5] Mallick-Searle T, Snodgrass B, Brant JM. Postherpetic neuralgia: epidemiology, pathophysiology, and pain management pharmacology. J Multidiscip Healthc. 2016;21(9):447–54. 10.2147/JMDH.S106340 PMC5036669 27703368

[6] Hagen EM, Rekand T. Management of neuropathic pain associated with spinal cord injury. Pain Ther. 2015;4(1):51–65. 25744501 10.1007/s40122-015-0033-y PMC4470971

[7] Lans J, Groot OQ, Hazewinkel MHJ, Kaiser PB, Lozano-Calderón SA, Heng M, et al. Factors related to neuropathic pain following lower extremity amputation. Plast Reconstr Surg. 2022;150(2):446–55. 35687412 10.1097/PRS.0000000000009334 PMC10375758

[8] Cortes-Altamirano JL, Reyes-Long S, Bandala C, Morraz-Varela A, Bonilla-Jaime H, Alfaro-Rodriguez A. Neuropathic pain in Parkinson’s disease. Neurol India. 2022;70(5):1879–86. 36352582 10.4103/0028-3886.359257

[9] Klit H, Finnerup NB, Jensen TS. Central post-stroke pain: clinical characteristics, pathophysiology, and management. Lancet Neurol. 2009;8(9):857–68. 19679277 10.1016/S1474-4422(09)70176-0

[10] Yawn BP, Wollan PC, Weingarten TN, Watson JC, Hooten WM, Melton LJ 3rd. The prevalence of neuropathic pain: clinical evaluation compared with screening tools in a community population. Pain Med. 2009;10(3):586–93. 20849570 10.1111/j.1526-4637.2009.00588.x PMC2964880

[11] Bouhassira D, Lantéri-Minet M, Attal N, Laurent B, Touboul C. Prevalence of chronic pain with neuropathic characteristics in the general population. Pain. 2008;136(3):380–7. 17888574 10.1016/j.pain.2007.08.013

[12] Torrance N, Smith BH, Bennett MI, Lee AJ. The epidemiology of chronic pain of predominantly neuropathic origin. Results from a general population survey. J Pain. 2006;7(4):281–9. 16618472 10.1016/j.jpain.2005.11.008

[13] Van Acker K, Bouhassira D, De Bacquer D, Weiss S, Matthys K, Raemen H, et al. Prevalence and impact on quality of life of peripheral neuropathy with or without neuropathic pain in type 1 and type 2 diabetic patients attending hospital outpatients clinics. Diabetes Metab. 2009;35(3):206–13. 19297223 10.1016/j.diabet.2008.11.004

[14] Daousi C, MacFarlane IA, Woodward A, Nurmikko TJ, Bundred PE, Benbow SJ. Chronic painful peripheral neuropathy in an urban community: a controlled comparison of people with and without diabetes. Diabet Med. 2004;21(9):976–82. 15317601 10.1111/j.1464-5491.2004.01271.x

[15] Abbott CA, Malik RA, Van Ross ER, Kulkarni J, Boulton AJ. Prevalence and characteristics of painful diabetic neuropathy in a large community-based diabetic population in the UK. Diabetes Care. 2011;34(10):2220–4. 21852677 10.2337/dc11-1108 PMC3177727

[16] Helgason S, Petursson G, Gudmundsson S, Sigurdsson JA. Prevalence of postherpetic neuralgia after a first episode of herpes zoster: prospective study with long term follow up. BMJ. 2000;321(7264):794. 11009518 10.1136/bmj.321.7264.794 PMC27491

[17] Bouhassira D, Chassany O, Gaillat J, Hanslik T, Launay O, Mann C, et al. Patient perspective on herpes zoster and its complications: an observational prospective study in patients aged over 50 years in general practice. Pain. 2012;153(2):342–9. 22138256 10.1016/j.pain.2011.10.026

[18] Kerba M, Wu JS, Duan Q, Hagen NA, Bennett MI. Neuropathic pain features in patients with bone metastases referred for palliative radiotherapy. J Clin Oncol. 2010;28(33):4892–7. 20921451 10.1200/JCO.2010.28.6559

[19] de Paredes MG, del Gonzalez Moral F, Del Prado PM, Ciriquián JM, Francés SE, Dols MC, et al. First evidence of oncologic neuropathic pain prevalence after screening 8615 cancer patients. Results of the On study. Ann Oncol. 2011;22(4):924–30. 20926548 10.1093/annonc/mdq449

[20] Bouhassira D, Luporsi E, Krakowski I. Prevalence and incidence of chronic pain with or without neuropathic characteristics in patients with cancer. Pain. 2017;158(6):1118–25. 28267066 10.1097/j.pain.0000000000000895

[21] Guastella V, Mick G, Soriano C, Vallet L, Escande G, Dubray C, et al. A prospective study of neuropathic pain induced by thoracotomy: incidence, clinical description, and diagnosis. Pain. 2011;152(1):74–81. 21075523 10.1016/j.pain.2010.09.004

[22] Gärtner R, Jensen MB, Nielsen J, Ewertz M, Kroman N, Kehlet H. Prevalence of and factors associated with persistent pain following breast cancer surgery. JAMA. 2009;302(18):1985–92. 19903919 10.1001/jama.2009.1568

[23] Kehlet H, Jensen TS, Woolf CJ. Persistent postsurgical pain: risk factors and prevention. Lancet. 2006;367(9522):1618–25. 16698416 10.1016/S0140-6736(06)68700-X

[24] Nikolajsen L. Postamputation pain: studies on mechanisms. Dan Med J. 2012;59(10):B4527. 23158899

[25] Poobalan AS, Bruce J, Smith WC, King PM, Krukowski ZH, Chambers WA. A review of chronic pain after inguinal herniorrhaphy. Clin J Pain. 2003;19(1):48–54. 12514456 10.1097/00002508-200301000-00006

[26] Dualé C, Ouchchane L, Schoeffler P; EDONIS Investigating Group, Dubray C. Neuropathic aspects of persistent postsurgical pain: a French multicenter survey with a 6-month prospective follow-up. J Pain. 2014;15(1):24.e1–20. 10.1016/j.jpain.2013.08.014 24373573

[27] Solaro C, Brichetto G, Amato MP, Cocco E, Colombo B, D’Aleo G, et al. The prevalence of pain in multiple sclerosis: a multicenter cross-sectional study. Neurology. 2004;63(5):919–21. 15365151 10.1212/01.wnl.0000137047.85868.d6

[28] Svendsen KB, Jensen TS, Overvad K, Hansen HJ, Koch-Henriksen N, Bach FW. Pain in patients with multiple sclerosis: a population-based study. Arch Neurol. 2003;60(8):1089–94. 12925364 10.1001/archneur.60.8.1089

[29] Osterberg A, Boivie J, Thuomas KA. Central pain in multiple sclerosis–prevalence and clinical characteristics. Eur J Pain. 2005;9(5):531–42. 16139182 10.1016/j.ejpain.2004.11.005

[30] Brochet B, Deloire MS, Ouallet JC, Salort E, Bonnet M, Jové J, et al. Pain and quality of life in the early stages after multiple sclerosis diagnosis: a 2-year longitudinal study. Clin J Pain. 2009;25(3):211–7. 19333171 10.1097/AJP.0b013e3181891347

[31] Siddall PJ, McClelland JM, Rutkowski SB, Cousins MJ. A longitudinal study of the prevalence and characteristics of pain in the first 5 years following spinal cord injury. Pain. 2003;103(3):249–57. 12791431 10.1016/S0304-3959(02)00452-9

[32] Siddall PJ, Taylor DA, McClelland JM, Rutkowski SB, Cousins MJ. Pain report and the relationship of pain to physical factors in the first 6 months following spinal cord injury. Pain. 1999;81(1–2):187–97. 10353507 10.1016/s0304-3959(99)00023-8

[33] Klit H, Finnerup NB, Andersen G, Jensen TS. Central poststroke pain: a population-based study. Pain. 2011;152(4):818–24. 21272999 10.1016/j.pain.2010.12.030

[34] Bouhassira D. Neuropathic pain: definition, assessment and epidemiology. Rev Neurol (Paris). 2019;175(1–2):16–25. 30385075 10.1016/j.neurol.2018.09.016

[35] Buhmann C, Wrobel N, Grashorn W, Fruendt O, Wesemann K, Diedrich S, et al. Pain in Parkinson disease: a cross-sectional survey of its prevalence, specifics, and therapy. J Neurol. 2017;264(4):758–69. 28243753 10.1007/s00415-017-8426-y

[36] Adoukonou T, Gnonlonfoun D, Kpozehouen A, Adjien C, Tchaou B, Tognon-Tchegnonsi F, et al. Prevalence and characteristics of chronic pain with neuropathic component at Parakou in northern Benin in 2012. Rev Neurol (Paris). 2014;170(11):703–11. 25444451 10.1016/j.neurol.2014.07.013

[37] de Moraes Vieira ÉB, Garcia JBS, da Silva AAM, Araújo RLTM, Jansen RCS. Prevalence, characteristics, and factors associated with chronic pain with and without neuropathic characteristics in São Luís. Brazil J Pain Symptom Manage. 2012;44(2):239–51. 22871508 10.1016/j.jpainsymman.2011.08.014

[38] Harifi G, Amine M, Ait Ouazar M, Boujemaoui A, Ouilki I, Rekkab I, et al. Prevalence of chronic pain with neuropathic characteristics in the Moroccan general population: a national survey. Pain Med. 2013;14(2):287–92. 23241023 10.1111/pme.12009

[39] Elzahaf RA, Johnson MI, Tashani OA. The epidemiology of chronic pain in Libya: a cross-sectional telephone survey. BMC Public Health. 2016;16:1–14. 27514513 10.1186/s12889-016-3349-6 PMC4982430

[40] Costigan M, Scholz J, Woolf CJ. Neuropathic pain: a maladaptive response of the nervous system to damage. Annu Rev Neurosci. 2009;32:1–32. 19400724 10.1146/annurev.neuro.051508.135531 PMC2768555

[41] Miclescu A, Straatmann A, Gkatziani P, Butler S, Karlsten R, Gordh T. Chronic neuropathic pain after traumatic peripheral nerve injuries in the upper extremity: prevalence, demographic and surgical determinants, impact on health and on pain medication. Scand J Pain. 2019;20(1):95–108. 31536038 10.1515/sjpain-2019-0111

[42] Campbell JN, Meyer RA. Mechanisms of neuropathic pain. Neuron. 2006;52(1):77–92. 17015228 10.1016/j.neuron.2006.09.021 PMC1810425

[43] Sommer C, Leinders M, Üçeyler N. Inflammation in the pathophysiology of neuropathic pain. Pain. 2018;159(3):595–602. 29447138 10.1097/j.pain.0000000000001122

[44] Finnerup NB, Kuner R, Jensen TS. Neuropathic pain: from mechanisms to treatment. Physiol Rev. 2021;101(1):259–301. 32584191 10.1152/physrev.00045.2019

[45] Kocot-Kępska M, Zajączkowska R, Mika J, Wordliczek J, Dobrogowski J, Przeklasa-Muszyńska A. Peripheral mechanisms of neuropathic pain-the role of neuronal and non-neuronal interactions and their implications for topical treatment of neuropathic pain. Pharmaceuticals (Basel). 2021;14(2):77. 33498496 10.3390/ph14020077 PMC7909513

[46] Ma YC, Kang ZB, Shi YQ, Ji WY, Zhou WM, Nan W. The complexity of neuropathic pain and central sensitization: exploring mechanisms and therapeutic prospects. J Integr Neurosci. 2024;23(5):89. 38812380 10.31083/j.jin2305089

[47] Schwartzman RJ, Grothusen J, Kiefer TR, Rohr P. Neuropathic central pain: epidemiology, etiology, and treatment options. Arch Neurol. 2001;58(10):1547–50. 11594911 10.1001/archneur.58.10.1547

[48] Myers RR, Campana WM, Shubayev VI. The role of neuroinflammation in neuropathic pain: mechanisms and therapeutic targets. Drug Discov Today. 2006;11(1–2):8–20. 16478686 10.1016/S1359-6446(05)03637-8

[49] Li XY, Wan Y, Tang SJ, Guan Y, Wei F, Ma D. Maladaptive plasticity and neuropathic pain. Neural Plast. 2016;2016:4842159. 26925270 10.1155/2016/4842159 PMC4748137

[50] Sindrup SH, Otto M, Finnerup NB, Jensen TS. Antidepressants in the treatment of neuropathic pain. Basic Clin Pharmacol Toxicol. 2005;96(6):399–409. 15910402 10.1111/j.1742-7843.2005.pto_96696601.x

[51] Tremont-Lukats IW, Megeff C, Backonja MM. Anticonvulsants for neuropathic pain syndromes: mechanisms of action and place in therapy. Drugs. 2000;60(5):1029–52. 11129121 10.2165/00003495-200060050-00005

[52] Sommer C, Cruccu G. Topical treatment of peripheral neuropathic pain: applying the evidence. J Pain Symptom Manage. 2017;53(3):614–29. 28042075 10.1016/j.jpainsymman.2016.09.015

[53] Smith HS. Opioids and neuropathic pain. Pain Physician. 2012;15(3):ES93-110. 22786465

[54] Gibson W, Wand BM, O’Connell NE. Transcutaneous electrical nerve stimulation (TENS) for neuropathic pain in adults. Cochrane Database Syst Rev. 2017;9(9):1011976. 10.1002/14651858.CD011976.pub2 PMC6426434 28905362

[55] Dones I, Levi V. Spinal cord stimulation for neuropathic pain: current trends and future applications. Brain Sci. 2018;8(8):138. 30042314 10.3390/brainsci8080138 PMC6119923

[56] Zhang YH, Hu HY, Xiong YC, Peng C, Hu L, Kong YZ, et al. Exercise for neuropathic pain: a systematic review and expert consensus. Front Med (Lausanne). 2021;24(8):756940. 10.3389/fmed.2021.756940 PMC8654102 34901069

[57] Pickering G. Analgesic use in the older person. Curr Opin Support Palliat Care. 2012;6(2):207–12. 22469664 10.1097/SPC.0b013e32835242d2

[58] Stompór M, Grodzicki T, Stompór T, Zaborowski P, Piechota M, Wójcik T, et al. Prevalence of chronic pain, particularly with neuropathic component, and its effect on overall functioning of elderly patients. Med Sci Monit Int Med J Exp Clin Res. 2019;25:2695–701. 10.12659/MSM.911260 PMC6475124 31018630

[59] Pickering G, Marcoux M, Chapiro S, et al. An algorithm for neuropathic pain management in older people. Drugs Aging. 2016;33(8):575–83. 27510615 10.1007/s40266-016-0389-7 PMC5012149

[60] Pedowitz EJ, Abrams RMC, Simpson DM. Management of neuropathic pain in the geriatric population. Clin Geriatr Med. 2021;37(2):361–76. 33858616 10.1016/j.cger.2021.01.008

[61] Giovannini S, Coraci D, Brau F, Galluzzo V, Loreti C, Caliandro P, et al. Neuropathic pain in the elderly. Diagnostics (Basel). 2021;11(4):613. 33808121 10.3390/diagnostics11040613 PMC8066049

[62] Torrance N, Ferguson JA, Afolabi E, Bennett MI, Serpell MG, Dunn KM, et al. Neuropathic pain in the community: more under-treated than refractory? Pain. 2013;154:690–9. 23485369 10.1016/j.pain.2012.12.022 PMC3630326

[63] Finnerup NB, Attal N, Haroutounian S, McNicol E, Baron R, Dworkin RH, et al. Pharmacotherapy for neuropathic pain in adults: a systematic review and meta-analysis. Lancet Neurol. 2015;14(2):162–73. 25575710 10.1016/S1474-4422(14)70251-0 PMC4493167

[64] Marzbani H, Marateb HR, Mansourian M. Neurofeedback: a comprehensive review on system design, methodology and clinical applications. Basic Clin Neurosci. 2016;7(2):143–58. 27303609 10.15412/J.BCN.03070208 PMC4892319

[65] Kopanska M, Ochojska D, Trojniak J, Sarzynska I, Szczygielski J. The role of quantitative electroencephalography in diagnostic workup of mental disorders. J Physiol Pharmacol. 2024;75(4). 10.26402/jpp.2024.4.02. 10.26402/jpp.2024.4.02 39415522

[66] Ros T, Baars BJ, Lanius RA, Vuilleumier P. Tuning pathological brain oscillations with neurofeedback: a systems neuroscience framework. Front Hum Neurosci. 2014;18(8):1008. 10.3389/fnhum.2014.01008 PMC4270171 25566028

[67] Dessy E, Mairesse O, van Puyvelde M, Cortoos A, Neyt X, Pattyn N. Train your brain? Can we really selectively train specific EEG frequencies with neurofeedback training. Front Hum Neurosci. 2020;10(14):22. 10.3389/fnhum.2020.00022 PMC7077336 32210777

[68] Kopańska M, Ochojska D, Sarzyńska I, Trojniak J, Banaś-Ząbczyk A, Szczygielski J. The use of quantitative electroencephalography (eyes closed) to assess the effectiveness of neurofeedback in therapy in children with mild autism spectrum disorders that reveal attention deficit disorders. Acta Neuropsychologica. 2025;23(1):27–46.

[69] Arns M, Clark CR, Trullinger M, deBeus R, Mack M, Aniftos M. Neurofeedback and attention-deficit/hyperactivity-disorder (ADHD) in children: rating the evidence and proposed guidelines. Appl Psychophysiol Biofeedback. 2020;45(2):39–48. 32206963 10.1007/s10484-020-09455-2 PMC7250955

[70] Chen C, Xiao X, Belkacem AN, Lu L, Wang X, Yi W, et al. Efficacy evaluation of neurofeedback-based anxiety relief. Front Neurosci. 2021;28(15):758068. 10.3389/fnins.2021.758068 PMC8581142 34776855

[71] Lee YJ, Lee GW, Seo WS, Koo BH, Kim HG, Cheon EJ. Neurofeedback treatment on depressive symptoms and functional recovery in treatment-resistant patients with major depressive disorder: an open-label pilot study. J Korean Med Sci. 2019;34(42):e287. 31674161 10.3346/jkms.2019.34.e287 PMC6823520

[72] Lambert-Beaudet F, Journault WG, Rudziavic Provençal A, Bastien CH. Neurofeedback for insomnia: current state of research. World J Psychiatry. 2021;11(10):897–914. 34733650 10.5498/wjp.v11.i10.897 PMC8546766

[73] Walker JE. QEEG-guided neurofeedback for recurrent migraine headaches. Clin EEG Neurosci. 2011;42(1):59–61. 21309444 10.1177/155005941104200112

[74] Panisch LS, Hai AH. The effectiveness of using neurofeedback in the treatment of post-traumatic stress disorder: a systematic review. Trauma Violence Abuse. 2020;21(3):541–50. 29890906 10.1177/1524838018781103

[75] Patel K, Sutherland H, Henshaw J, Taylor JR, Brown CA, Casson AJ, et al. Effects of neurofeedback in the management of chronic pain: a systematic review and meta-analysis of clinical trials. Eur J Pain. 2020;24(8):1440–57. 32502283 10.1002/ejp.1612

[76] Schuurman BB, Lousberg RL, Schreiber JU, van Amelsvoort TAMJ, Vossen CJ. A Scoping review of the effect of EEG Neurofeedback on pain complaints in adults with chronic pain. J Clin Med. 2024;13(10):2813. 38792353 10.3390/jcm13102813 PMC11122542

[77] Diotaiuti P, Corrado S, Tosti B, Spica G, Di Libero T, D’Oliveira A, et al. Evaluating the effectiveness of neurofeedback in chronic pain management: a narrative review. Front Psychol. 2024;6(15):1369487. 10.3389/fpsyg.2024.1369487 PMC11104502 38770259

[78] Roy R, de la Vega R, Jensen MP, Miró J. Neurofeedback for pain management: a systematic review. Front Neurosci. 2020;16(14):671. 10.3389/fnins.2020.00671 PMC7378966 32765208

[79] Sarnthein J, Stern J, Aufenberg C, Rousson V, Jeanmonod D. Increased EEG power and slowed dominant frequency in patients with neurogenic pain. Brain. 2006;129(Pt 1):55–64. 16183660 10.1093/brain/awh631

[80] Boord P, Siddall PJ, Tran Y, Herbert D, Middleton J, Craig A. Electroencephalographic slowing and reduced reactivity in neuropathic pain following spinal cord injury. Spinal Cord. 2008;46:118–23. 17502876 10.1038/sj.sc.3102077

[81] Jensen MP, Sherlin LH, Gertz KJ, Braden AL, Kupper AE, Gianas A, et al. Brain EEG activity correlates of chronic pain in persons with spinal cord injury: clinical implications. Spinal Cord. 2013;51(1):55–8. 22801188 10.1038/sc.2012.84

[82] Stern J, Jeanmonod D, Sarnthein J. Persistent EEG overactivation in the cortical pain matrix of neurogenic pain patients. Neuroimage. 2006;31(2):721–31. 16527493 10.1016/j.neuroimage.2005.12.042

[83] Michels L, Moazami-Goudarzi M, Jeanmonod D. Correlations between EEG and clinical outcome in chronic neuropathic pain: surgical effects and treatment resistance. Brain Imaging Behav. 2011;5:329–48. 21948245 10.1007/s11682-011-9135-2

[84] Vuckovic A, Gallardo VJF, Jarjees M, Fraser M, Purcell M. Prediction of central neuropathic pain in spinal cord injury based on EEG classifier. Clin Neurophysiol. 2018;129(8):1605–17. 29886266 10.1016/j.clinph.2018.04.750

[85] Wydenkeller S, Maurizio S, Dietz V, Halder P. Neuropathic pain in spinal cord injury: significance of clinical and electrophysiological measures. Eur J Neurosci. 2009;30(1):91–9. 19558605 10.1111/j.1460-9568.2009.06801.x

[86] Di Pietro F, Macey PM, Rae CD, Alshelh Z, Macefield VG, Vickers ER, et al. The relationship between thalamic GABA content and resting cortical rhythm in neuropathic pain. Hum Brain Mapp. 2018;39(5):1945–56. 29341331 10.1002/hbm.23973 PMC6866606

[87] Zhou R, Wang J, Qi W, Liu FY, Yi M, Guo H, et al. Elevated resting state gamma oscillatory activities in electroencephalogram of patients with post-herpetic neuralgia. Front Neurosci. 2018;23(12):750. 10.3389/fnins.2018.00750 PMC6205978 30405337

[88] Krupina NA, Churyukanov MV, Kukushkin ML, Yakhno NN. Central neuropathic pain and profiles of quantitative electroencephalography in multiple sclerosis patients. Front Neurol. 2020;21(10):1380. 10.3389/fneur.2019.01380 PMC6990108 32038459

[89] Levitt J, Edhi MM, Thorpe RV, Leung JW, Michishita M, Koyama S, et al. Pain phenotypes classified by machine learning using electroencephalography features. Neuroimage. 2020;223:117256. 32871260 10.1016/j.neuroimage.2020.117256 PMC9084327

[90] Teixeira M, Mancini C, Wicht CA, Maestretti G, Kuntzer T, Cazzoli D, et al. Beta electroencephalographic oscillation is a potential GABAergic biomarker of chronic peripheral neuropathic pain. Front Neurosci. 2021;26(15):594536. 10.3389/fnins.2021.594536 PMC7952534 33716642

[91] Meneses FM, Queirós FC, Montoya P, Miranda JG, Dubois-Mendes SM, Sá KN, et al. Patients with rheumatoid arthritis and chronic pain display enhanced alpha power density at rest. Front Hum Neurosci. 2016;4(10):395. 10.3389/fnhum.2016.00395 PMC4972828 27540360

[92] Zolezzi DM, Alonso-Valerdi LM, Ibarra-Zarate DI. EEG frequency band analysis in chronic neuropathic pain: a linear and nonlinear approach to classify pain severity. Comput Methods Programs Biomed. 2023;230:107349. 36689806 10.1016/j.cmpb.2023.107349

[93] Wang D, Zhang X, Xin C, Wang C, Yue S, Guo D, et al. Electroencephalography-based biological and functional characteristics of spinal cord injury patients with neuropathic pain and numbness. Front Neurosci. 2024;1(18):1356858. 10.3389/fnins.2024.1356858 PMC11094546 38751860

[94] Rajan J, Gaur GS, Shanmugavel KSA. Relation between heart rate variability and spectral analysis of electroencephalogram in chronic neuropathic pain patients. Korean J Physiol Pharmacol. 2024;28(3):253–64. 38682173 10.4196/kjpp.2024.28.3.253 PMC11058544

[95] van den Broeke EN, Wilder-Smith OH, van Goor H, Vissers KC, van Rijn CM. Patients with persistent pain after breast cancer treatment show enhanced alpha activity in spontaneous EEG. Pain Med. 2013;14(12):1893–9. 24034712 10.1111/pme.12216

[96] Groh A, Krieger P, Mease RA, Henderson L. Acute and chronic pain processing in the thalamocortical system of humans and animal models. Neuroscience. 2018;1(387):58–71. 10.1016/j.neuroscience.2017.09.042 28978414

[97] Sarnthein J, Jeanmonod D. High thalamocortical theta coherence in patients with neurogenic pain. Neuroimage. 2008;39(4):1910–7. 18060808 10.1016/j.neuroimage.2007.10.019

[98] Llinás RR, Ribary U, Jeanmonod D, Kronberg E, Mitra PP. Thalamocortical dysrhythmia: a neurological and neuropsychiatric syndrome characterized by magnetoencephalography. Proc Natl Acad Sci USA. 1999;96:15222–7. 10.1073/pnas.96.26.15222 PMC24801 10611366

[99] Walton KD, Llinás RR. Central pain as a thalamocortical dysrhythmia: a thalamic efference disconnection? In: Kruger L, Light AR, editors. Translational pain research: from mouse to man. Boca Raton (FL): CRC Press/Taylor & Francis; 2010. Chapter 13. 21882456

[100] Xia C, Zhou J, Lu C, Wang Y, Tang T, Cai Y, et al. Characterizing diaschisis-related thalamic perfusion and diffusion after middle cerebral artery infarction. Stroke. 2021;52:2319–27. 33971741 10.1161/STROKEAHA.120.032464

[101] Pinheiro ES, de Queirós FC, Montoya P, Santos CL, do Nascimento MA, Ito CH, et al. Electroencephalographic patterns in chronic pain: a systematic review of the literature. PLoS One. 2016;11(2):e0149085. 26914356 10.1371/journal.pone.0149085 PMC4767709

[102] Vanneste S, Song JJ, De Ridder D. Thalamocortical dysrhythmia detected by machine learning. Nat Commun. 2018;9:1103. 29549239 10.1038/s41467-018-02820-0 PMC5856824

[103] Cauda F, Sacco K, D’Agata F, Duca S, Cocito D, Geminiani G, et al. Low-frequency BOLD fluctuations demonstrate altered thalamocortical connectivity in diabetic neuropathic pain. BMC Neurosci. 2009;10:138. 19941658 10.1186/1471-2202-10-138 PMC2789078

[104] Tu Y, Fu Z, Mao C, et al. Distinct thalamocortical network dynamics are associated with the pathophysiology of chronic low back pain. Nat Commun. 2020;11:3948. 32769984 10.1038/s41467-020-17788-z PMC7414843

[105] Choi S, Yu E, Hwang E, Llinás RR. Pathophysiological implication of CaV3.1 T-type Ca2+ channels in trigeminal neuropathic pain. Proc Natl Acad Sci USA. 2016;113(8):2270–5. 26858455 10.1073/pnas.1600418113 PMC4776481

[106] Alshelh Z, Di Pietro F, Youssef AM, Reeves JM, Macey PM, Vickers ER, et al. Chronic neuropathic pain: it’s about the rhythm. J Neurosci. 2016;36(3):1008–18. 26791228 10.1523/JNEUROSCI.2768-15.2016 PMC6602000

[107] Henderson LA, Peck CC, Petersen ET, et al. Chronic pain: lost inhibition? J Neurosci. 2013;33(17):7574–82. 23616562 10.1523/JNEUROSCI.0174-13.2013 PMC6619566

[108] Asadauskas A, Stieger A, Luedi MM, Andereggen L. Advancements in modern treatment approaches for central post-stroke pain: a narrative review. J Clin Med. 2024;13(18):5377. 39336863 10.3390/jcm13185377 PMC11432561

[109] Shih HC, Kuan YH, Shyu BC. Targeting brain-derived neurotrophic factor in the medial thalamus for the treatment of central poststroke pain in a rodent model. Pain. 2017;158(7):1302–13. 28394853 10.1097/j.pain.0000000000000915 PMC5472007

[110] Nawaz R, Suen H, Ullah R, et al. Electroencephalography longitudinal markers of central neuropathic pain intensity in spinal cord injury: a home-based pilot study. Biomedicines. 2024;12(12):2751. 39767658 10.3390/biomedicines12122751 PMC11672874

[111] Gustin SM, Wrigley PJ, Youssef AM, et al. Thalamic activity and biochemical changes in individuals with neuropathic pain after spinal cord injury. Pain. 2014;155(5):1027–36. 24530612 10.1016/j.pain.2014.02.008 PMC4410007

[112] Jones EG. Thalamocortical dysrhythmia and chronic pain. Pain. 2010;150(1):4–5. 20395046 10.1016/j.pain.2010.03.022

[113] Lőrincz ML, Crunelli V, Hughes SW. Cellular dynamics of cholinergically induced α (8–13 hz) rhythms in sensory thalamic nuclei in vitro. J Neurosci. 2008;28(3):660–71. 18199766 10.1523/JNEUROSCI.4468-07.2008 PMC2778076

[114] Vijayan S, Kopell NJ. Thalamic model of awake alpha oscillations and implications for stimulus processing. Proc Natl Acad Sci U S A. 2012;109(45):18553–8. 23054840 10.1073/pnas.1215385109 PMC3494906

[115] Lukashevich IP, Sazonova OB. The effect of lesions of different parts of the optic thalamus on the nature of the bioelectrical activity of the human brain. Zh Vyssh Nerv Deiat Im I P Pavlova. 1996 Sep-Oct;46(5):866–74. [Russian] 9054138

[116] Pires MP, McBenedict B, Ahmed IE, Yau RCC, Fong YB, Goh KS, et al. Exploring the thalamus as a target for neuropathic pain management: an integrative review. Cureus. 2024;16(5):e60130. 38864037 10.7759/cureus.60130 PMC11165437

[117] Gustin SM, Peck CC, Wilcox SL, Nash PG, Murray GM, Henderson LA. Different pain, different brain: thalamic anatomy in neuropathic and non-neuropathic chronic pain syndromes. J Neurosci. 2011;31:5956–64. 21508220 10.1523/JNEUROSCI.5980-10.2011 PMC6632967

[118] Gustin SM, Wrigley PJ, Siddall PJ, Henderson LA. Brain anatomy changes associated with persistent neuropathic pain following spinal cord injury. Cereb Cortex. 2010;20:1409–19. 19815621 10.1093/cercor/bhp205

[119] Jones SR, Pinto DJ, Kaper TJ, Kopell N. Alpha-frequency rhythms desynchronize over long cortical distances: a modeling study. J Comput Neurosci. 2000;9(3):271–91. 11139043 10.1023/a:1026539805445

[120] Lőrincz ML, Kékesi KA, Juhász G, Crunelli V, Hughes SW. Temporal framing of thalamic relay-mode firing by phasic inhibition during the alpha rhythm. Neuron. 2009;63(5):683–96. 19755110 10.1016/j.neuron.2009.08.012 PMC2791173

[121] Kim JA, Davis KD. Neural oscillations: understanding a neural code of pain. Neuroscientist. 2021;27(5):544–70. 32981457 10.1177/1073858420958629

[122] Yowtak J, Wang J, Kim HY, Lu Y, Chung K, Chung JM. Effect of antioxidant treatment on spinal GABA neurons in a neuropathic pain model in the mouse. Pain. 2013;154:2469–76. 23880056 10.1016/j.pain.2013.07.024 PMC3816493

[123] Moon HC, Park YS. Reduced GABAergic neuronal activity in zona incerta causes neuropathic pain in a rat sciatic nerve chronic constriction injury model. J Pain Res. 2017;10:1125–34. 28546770 10.2147/JPR.S131104 PMC5436785

[124] Ju YH, Cho J, Park JY, et al. Tonic excitation by astrocytic GABA causes neuropathic pain by augmenting neuronal activity and glucose metabolism. Exp Mol Med. 2024;56:1193–205. 38760512 10.1038/s12276-024-01232-z PMC11148027

[125] Peng W, Babiloni C, Mao Y, Hu Y. Subjective pain perception mediated by alpha rhythms. Biol Psychol. 2015;109:141–50. 26026894 10.1016/j.biopsycho.2015.05.004

[126] Mazaheri A, Seminowicz DA, Furman AJ. Peak alpha frequency as a candidate biomarker of pain sensitivity: the importance of distinguishing slow from slowing. Neuroimage. 2022;15(262):119560. 10.1016/j.neuroimage.2022.119560 35973563

[127] Hassan MA, Fraser M, Conway BA, Allan DB, Vuckovic A. The mechanism of neurofeedback training for treatment of central neuropathic pain in paraplegia: a pilot study. BMC Neurol. 2015;13(15):200. 10.1186/s12883-015-0445-7 PMC4604632 26462651

[128] Hasan MA, Vuckovic A, Qazi SA, Yousuf Z, Shahab S, Fraser M. Immediate effect of neurofeedback training on the pain matrix and cortical areas involved in processing neuropsychological functions. Neurol Sci. 2021;42(11):4551–61. 33624179 10.1007/s10072-021-05125-1

[129] Vučković A, Altaleb MKH, Fraser M, McGeady C, Purcell M. EEG correlates of self-managed neurofeedback treatment of central neuropathic pain in chronic spinal cord injury. Front Neurosci. 2019;25(13):762. 10.3389/fnins.2019.00762 PMC6670070 31404253

[130] Anil K, Demain S, Burridge J, Simpson D, Taylor J, Cotter I, Vuckovic A. The importance of self-efficacy and negative affect for neurofeedback success for central neuropathic pain after a spinal cord injury. Sci Rep. 2022;12(1):10949. 35768524 10.1038/s41598-022-15213-7 PMC9243249

[131] Hasan MA, Fraser M, Conway BA, Allan DB, Vučković A. Reversed cortical over-activity during movement imagination following neurofeedback treatment for central neuropathic pain. Clin Neurophysiol. 2016;127(9):3118–27. 27472548 10.1016/j.clinph.2016.06.012 PMC4988467

[132] Al-Taleb MKH, Purcell M, Fraser M, Petric-Gray N, Vuckovic A. Home used, patient self-managed, brain-computer interface for the management of central neuropathic pain post spinal cord injury: usability study. J Neuroeng Rehabil. 2019;16(1):128. 31666096 10.1186/s12984-019-0588-7 PMC6822418

[133] Hasan M, Fraser M, Qazi S. Quantitative criteria for validation of electroencephalogram neurofeedback training protocol: a pilot study on neuropathic pain. NED Univ J Res. 2019;XVI:67–79.

[134] Vuckovic A, Jajrees M, Purcell M, Berry H, Fraser M. Electroencephalographic predictors of neuropathic pain in subacute spinal cord injury. J Pain. 2018;19(11):1256.e1-1256.e17. 29751110 10.1016/j.jpain.2018.04.011

[135] Ishii R, Canuet L, Aoki Y, Hata M, Iwase M, Ikeda S, et al. Healthy and pathological brain aging: from the perspective of oscillations, functional connectivity, and signal complexity. Neuropsychobiology. 2017;75(4):151–61. 29466802 10.1159/000486870

[136] Aird RB, Gastaut Y. Occipital and posterior electroencephalographic ryhthms. Electroencephalogr Clin Neurophysiol. 1959;11:637–56. 13792196 10.1016/0013-4694(59)90104-x

[137] Gil-Nagel A, Parra J, Irirarte J, Kanner A. Manual de electroencefalografía. Madrid: Mc Graw Hill Interamericana; 2002.

[138] van der Hiele K, Bollen ELEM, Vein AA, Reijntjes RHAM, Westendorp RGJ, van Buchem MA, et al. EEG markers of future cognitive performance in the elderly. J Clin Neurophysiol. 2008;25:83–9. 18340274 10.1097/WNP.0b013e31816a5b25

[139] Smailovic U, Koenig T, Kåreholt I, Andersson T, Kramberger MG, Winblad B, et al. Quantitative EEG power and synchronization correlate with Alzheimer’s disease CSF biomarkers. Neurobiol Aging. 2018;63:88–95. 29245058 10.1016/j.neurobiolaging.2017.11.005

[140] Prichep LS, John ER, Ferris SH, Reisberg B, Almas M, Alper K, et al. Quantitative EEG correlates of cognitive deterioration in the elderly. Neurobiol Aging. 1994;15:85–90. 8159266 10.1016/0197-4580(94)90147-3

[141] van der Hiele K, Vein AA, Reijntjes RHAM, Westendorp RGJ, Bollen ELEM, van Buchem MA, et al. EEG correlates in the spectrum of cognitive decline. Clin Neurophysiol. 2007;118:1931–9. 17604688 10.1016/j.clinph.2007.05.070

[142] Musaeus CS, Engedal K, Høgh P, Jelic V, Mørup M, Naik M, et al. EEG theta power is an early marker of cognitive decline in dementia due to Alzheimer’s disease. J Alzheimers Dis. 2018;64:1359–71. 29991135 10.3233/JAD-180300

[143] Roca-Stappung M, Fernández T, Becerra J, Mendoza-Montoya O, Espino M, Harmony T. Healthy aging: relationship between quantitative electroencephalogram and cognition. Neurosci Lett. 2012;510:115–20. 22266305 10.1016/j.neulet.2012.01.015

[144] Alatorre-Cruz GC, Fernández T, Castro-Chavira SA, González-López M, Sánchez-Moguel SM, Silva-Pereyra J. One-year follow-up of healthy older adults with electroencephalographic risk for neurocognitive disorder after neurofeedback training. J Alzheimers Dis. 2022;85(4):1767–81. 34974435 10.3233/JAD-215538 PMC8925127

[145] Laborda-Sánchez F, Cansino S. The effects of neurofeedback on aging-associated cognitive decline: a systematic review. Appl Psychophysiol Biofeedback. 2021;46(1):1–10. 33389281 10.1007/s10484-020-09497-6

[146] Yeh WH, Hsueh JJ, Shaw FZ. Neurofeedback of alpha activity on memory in healthy participants: a systematic review and meta-analysis. Front Hum Neurosci. 2021;5(14):562360. 10.3389/fnhum.2020.562360 PMC7813983 33469422

[147] Diaz MM, Caylor J, Strigo I, Lerman I, Henry B, Lopez E, et al. Toward composite pain biomarkers of neuropathic pain-focus on peripheral neuropathic pain. Front Pain Res (Lausanne). 2022;11(3):869215. 10.3389/fpain.2022.869215 PMC9130475 35634449

[148] Nwagwu CD, Sarris C, Tao YX, Mammis A. Biomarkers for chronic neuropathic pain and their potential application in spinal cord stimulation: a review. Transl Perioper Pain Med. 2016;1(3):33–8. 28480314 PMC5415348

[149] Thakkar B, Acevedo EO. BDNF as a biomarker for neuropathic pain: consideration of mechanisms of action and associated measurement challenges. Brain Behav. 2023;13(3):e2903. 36722793 10.1002/brb3.2903 PMC10013954

[150] Hamdan A, Galvez R, Katati M. Shedding light on neuropathic pain: current and emerging tools for diagnosis, screening, and quantification. SAGE Open Med. 2024;9(12):20503121231218984. 10.1177/20503121231218985 PMC10858674 38343869

[151] Sisignano M, Lötsch J, Parnham MJ, Geisslinger G. Potential biomarkers for persistent and neuropathic pain therapy. Pharmacol Ther. 2019;199:16–29. 30759376 10.1016/j.pharmthera.2019.02.004

